# Minimally Invasive Infrared Imaging of *Caenorhabditis elegans* Enabled by Reversible Chemical Immobilization and Second-Derivative Spectral Mapping

**DOI:** 10.1021/acsomega.6c03485

**Published:** 2026-08-26

**Authors:** Nodoka Wakita, Akiko Ito, Ryu Sato, Akihisa Terakita, Tatsuro Nishikino, Mitsumasa Koyanagi, Yuji Furutani

**Affiliations:** † Department of Life Science and Applied Chemistry, 12982 Nagoya Institute of Technology, Showa-ku, Nagoya 466-8555, Japan; ‡ Department of Biology, Graduate School of Science, Osaka Metropolitan University, 3-3-138 Sugimoto, Sumiyoshi-ku, Osaka 558-8585, Japan; § The OMU Advanced Research Institute for Natural Science and Technology, Osaka Metropolitan University, 3-3-138 Sugimoto, Sumiyoshi-ku, Osaka 558-8585, Japan; ∥ OptoBioTechnology Research Center, Nagoya Institute of Technology, Showa-ku, Nagoya 466-8555, Japan

## Abstract

The nematode *Caenorhabditis elegans* is a widely used model organism, yet its infrared (IR) spectroscopic characterization has been limited by motion and strong water absorption in the mid-infrared region. Here, we establish a minimally invasive mid-infrared imaging method for *C. elegans* by combining reversible immobilization with sodium azide (NaN_3_) and measurements in deuterium oxide (D_2_O). NaN_3_ effectively suppressed locomotion without inducing irreversible physiological damage, enabling high-quality acquisition of IR images. Mid-IR hyperspectral data were analyzed to extract lipid- and protein-specific bands for each pixel, followed by second-derivative analysis of the Amide I band to resolve protein secondary-structure components. This allowed decomposition of α-helical (1658 cm^–1^) and β-sheet (1628 cm^–1^) components and visualization of their spatial distributions *in vivo*. Notably, the 1628 cm^–1^/1658 + 1628 cm^–1^ ratio was significantly lower in the anterior region of the head, indicating region-specific differences in protein composition. Following imaging, worms recovered normal locomotion and retained reproductive capability, demonstrating that the method is minimally invasive and reversible. Overall, this work establishes a quantitative IR imaging framework for analyzing protein structure in living organisms at the micrometer scale. The combination of reversible immobilization and second-derivative spectral narrowing provides a powerful approach for investigating spatial variations in biomolecular structure *in vivo*.

## Graphical abstract

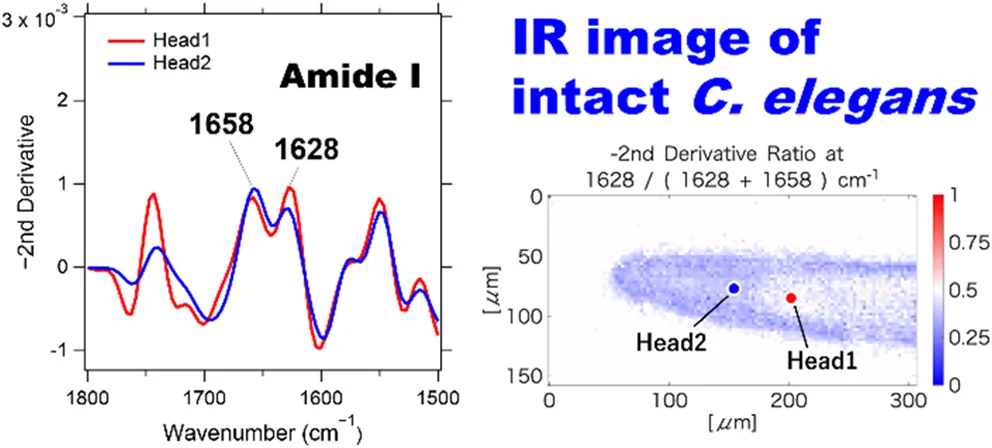

## 1 Introduction

The nematode *Caenorhabditis elegans* is a powerful model organism widely used in genetics, neurobiology, and developmental biology.^1–4^ Its small size, optical transparency, well-annotated cell lineage, and ease of genetic manipulation make it ideal for dissecting cellular and molecular processes *in vivo*. Fluorescence microscopy has played a central role in *C. elegans* research, enabling subcellular imaging with high spatial resolution.^5–7^ However, fluorescence-based approaches require the introduction of labels or genetically encoded probes, which can perturb native molecular environments and restrict observations to preselected molecular targets. Thus, a complementary, label-free imaging method capable of capturing endogenous biomolecular information is highly desirable.

Vibrational spectroscopic techniques, including Raman and infrared (IR) microspectroscopy, provide intrinsic chemical contrast without the need for exogenous probes.^8–14^ Raman microscopy has been applied to living organisms, including *C. elegans*, but it requires high-intensity visible or near-infrared laser irradiation. Such strong illumination can cause photodamage or heating, particularly in organisms with limited capacity for heat dissipation, and often limits the duration or sensitivity of live-cell observations. Even moderate increases in temperature, well below lethal levels, have been reported to influence developmental pathways and behavior in *C. elegans*, including the induction of stress-resistant dauer larvae^15,16^ and alterations in thermotaxis.^17–19^ These concerns highlight the need for an alternative label-free approach that minimizes phototoxicity while providing molecularly specific information.

Midinfrared (mid-IR) imaging is a promising solution because IR radiation interacts with fundamental vibrational modes of biomolecules while causing minimal light-induced damage. IR hyperspectral imaging enables direct visualization of proteins, lipids, nucleic acids, and metabolites by measuring their characteristic absorption bands, offering a comprehensive view of biochemical composition *in vivo*. Despite these advantages, mid-IR imaging has several inherent limitations, including spatial resolution constrained to several micrometers by the relatively long wavelengths of mid-IR radiation, as well as signal attenuation in optically thick samples, typically restricting practical sample thicknesses to the range of a few to several tens of micrometers. In addition, most previous mid-IR studies of *C. elegans* have been performed using dried, fixed, or otherwise irreversibly immobilized samples, avoiding complications associated with water absorption and organismal motion.^9,10,13^ As a result, IR imaging of *C. elegans* in the living state remains extremely limited. Recent work using quantum cascade lasers (QCLs) has demonstrated high-speed IR video imaging of moving worms;^8^ however, QCL systems typically cover only a narrow spectral region and do not provide full mid-IR hyperspectral information simultaneously—such as Amide I band (∼1650 cm^–1^) probing the protein secondary structures and C–H stretching vibrations (∼2900 cm^–1^) probing lipid molecules.^20,21^

To enable mid-IR imaging using an FT-IR microscope equipped with a focal plane array (FPA) detector and a step-scan technique covering a broad spectral range (3850–750 cm^–1^), two technical challenges must be addressed: (i) the strong absorption of liquid water that obscures critical spectral regions, and (ii) the continuous movement of live worms during image acquisition. In the present study, we overcame the first challenge by substituting H_2_O with D_2_O, which shifts O–H bending and stretching absorption away from the protein Amide I and the C–H stretching regions, respectively, enabling accurate analysis of protein and lipid bands. To address the second challenge, we employed sodium azide (NaN_3_), which rapidly and reversibly immobilizes worms by inhibiting mitochondrial respiration.^1^ NaN_3_ at an optimized concentration (0.1 M) halts worm locomotion long enough to acquire high-quality IR images while allowing full recovery of movement and reproduction after measurements. This reversible immobilization is essential for ensuring that the imaging process does not irreversibly compromise physiological viability.

By combining D_2_O-based sample preparation, reversible NaN_3_ immobilization, and FPA-based hyperspectral IR imaging, we established a method capable of acquiring full mid-IR spectra (including the Amide I and C–H stretching region) from intact *C. elegans* within minutes. Importantly, second-derivative analysis of pixel-resolved spectra enabled the separation of overlapping components corresponding to α-helical and β-sheet structures, allowing direct visualization of spatial variations in protein secondary structure within the worm body. Magnified analysis of the head region further revealed characteristic low β-sheet content in pharyngeal muscle tissues and peripheral cuticular structures, demonstrating that this method can resolve fine-scale molecular architecture *in vivo*.

This work represents a significant methodological advance toward truly label-free, minimally invasive vibrational imaging of intact multicellular organisms. In the future, integrating this approach with optogenetic stimulation, neuronal activation assays, or developmental studies may provide new opportunities to investigate dynamic molecular processes in *C. elegans* at the level of intrinsic biochemical signatures. Our results thus establish a foundational platform for exploring functional mechanisms in living systems using infrared chemical imaging.

## 2 Materials and Methods

### 2.1 Preparation of Buffer and Salt Solutions

#### 2.1.1 1 M Potassium Phosphate Buffer (pH 6.0)

KH_2_PO_4_ (54.15 g) and K_2_HPO_4_ (17.8 g) were dissolved in ∼400 mL of ultrapure water with stirring and gentle heating. The pH was adjusted to 6.0 using 8 M KOH, and the solution was brought to a final volume of 500 mL. The buffer was autoclaved (121 °C, 20 min) and used after cooling to room temperature.

#### 2.1.2 1 M MgSO_4_ Solution

MgSO_4_·7H_2_O (12.32 g) was dissolved in ∼40 mL of ultrapure water with stirring and adjusted to a final volume of 50 mL. The solution was autoclaved (121 °C, 20 min) and used after cooling.

#### 2.1.3 1 M CaCl_2_ Solution

CaCl_2_·2H_2_O (7.35 g) was dissolved in ∼40 mL of ultrapure water with stirring and adjusted to 50 mL. The solution was autoclaved (121 °C, 20 min) and used after cooling.

#### 2.1.4 LB Medium

Tryptone (10 g), yeast extract (5 g), and NaCl (10 g) were dissolved in ultrapure water and adjusted to 1 L. The medium was autoclaved (121 °C, 20 min) and cooled before use.

#### 2.1.5 *Escherichia coli* OP50 Culture

Glycerol stock–derived OP50 cells were inoculated into 3 mL of LB medium supplemented with streptomycin (1.5 μL of 0.2 g/mL stock) and incubated at 37 °C with shaking (180 rpm) for 16–18 h. The cultured *E. coli* OP50 was used as the food source for *C. elegans* during routine cultivation prior to the infrared imaging experiments.^1^

#### 2.1.6 NGM Plates

NaCl (1.5 g), Bacto Peptone (1.25 g), agar (7.5 g), cholesterol (0.5 mL of 5 mg/mL), and ultrapure water (500 mL) were autoclaved (121 °C, 20 min). After cooling to approximately 60 °C, the following sterilized supplements were added: 1 M potassium phosphate buffer (pH 6.0; 12.5 mL), 1 M MgSO_4_ (0.5 mL), 1 M CaCl_2_ (0.5 mL), streptomycin (150 μL of 0.2 g/mL), and nystatin (0.5 mL of 10 mg/mL). Approximately 8 mL of medium was dispensed into each 60 mm-diameter dish and allowed to solidify.

#### 2.1.7 OP50-Seeded NGM Plates

Cultured OP50 was spread onto NGM plates (two drops using a P1000 pipet) and dried overnight. Plates were sealed with Parafilm and stored at 20 °C until use.

#### 2.1.8 M9 Buffer (H_2_O)

Na_2_HPO_4_ (1.2 g), KH_2_PO_4_ (0.6 g), and NaCl (1.0 g) were dissolved in ultrapure water and adjusted to a final volume of 200 mL. The buffer was autoclaved (121 °C, 20 min). After cooling, the following sterilized supplements were added: 1 M MgSO_4_ (0.2 mL) and 2% gelatin (2 mL) solution. The H_2_O-based M9 buffer had a pH of 7.0.

#### 2.1.9 M9 Buffer (D_2_O)

Na_2_HPO_4_ (0.3 g), KH_2_PO_4_ (0.15 g), and NaCl (0.25 g) were dissolved in D_2_O and adjusted to a final volume of 5 mL (×10 solution). The ×10 solution was filtrated with 0.2 μm mesh. Then, 2 mL of the ×10 solution was diluted with D_2_O (18 mL) and 1 M MgSO_4_ in D_2_O solution (0.02 mL). Finally, the mixed solution was filtrated with 0.2 μm mesh. The D_2_O-based M9 buffer gave an apparent pH reading of 7.1 using a conventional glass electrode, corresponding to an estimated pD of 7.5 after applying the standard correction (+0.4).^22^

#### 2.1.10 0.1 M NaN_3_ Solution

NaN_3_ (0.0325 g) was dissolved in 5 mL of M9 buffer (D_2_O).

#### 2.1.11 5 mM levamisole solution

levamisole hydrochloride (6.02 mg) was dissolved in 5 mL of M9 buffer (D_2_O).

### 2.2 Worm Strains and Culture

*C. elegans* strain N2 (Allele name: tm4975), which lacks lite-1 encoding a native UV light photoreceptor and is therefore insensitive to visible and ultraviolet light, was obtained from the National BioResource Project (NBRP), Japan. Worms were routinely maintained at 20 °C on 60 mm NGM plates seeded with *E. coli* OP50 under standard culture conditions.^1^

### 2.3 Sample Preparation for IR Imaging

For infrared imaging experiments, six *C. elegans* individuals were transferred onto freshly prepared NGM plates seeded with *E. coli* OP50 and maintained at 20 °C. Infrared measurements were performed 3 days after transfer to the new plate (e.g., the worms transferred on Monday and IR imaging was performed on Thursday). A 25 mm-diameter, 2 mm thick CaF_2_ window was loaded with 4 μL of 0.1 M NaN_3_ (or 5 mM levamisole) in M9 buffer prepared with D_2_O. A large number of worms were obtained from eggs laid by the six transferred individuals, and worms with a body length of approximately 1 mm were selected for experiments, corresponding to the young adult to adult stage according to the WormAtlas classification.^23^ Among them, four worms were transferred onto the window. Three aluminum-foil spacers (20 μm thickness, 2.5 × 5 mm) were placed along the window edge, and a second CaF_2_ window was placed on top (Figure 1A and 1B). The assembly was sealed with Parafilm (Figure 1C), fixed onto the sample stage with screws, and used for IR measurements (Figure 1D). Sample preparation required approximately 10 min, including transfer of the worms onto the CaF_2_ window, placement of spacers, and sealing of the sample chamber. Under the optimized acquisition conditions used in this study, infrared imaging required an additional 6 min 47 s (64 scans, 8.0 cm^–1^ resolution), resulting in a total exposure time to D_2_O and NaN_3_ of approximately 17 min. A blank CaF_2_ window was used to acquire background spectra. The details are described in Results and Discussion section.

**Figure 1:**
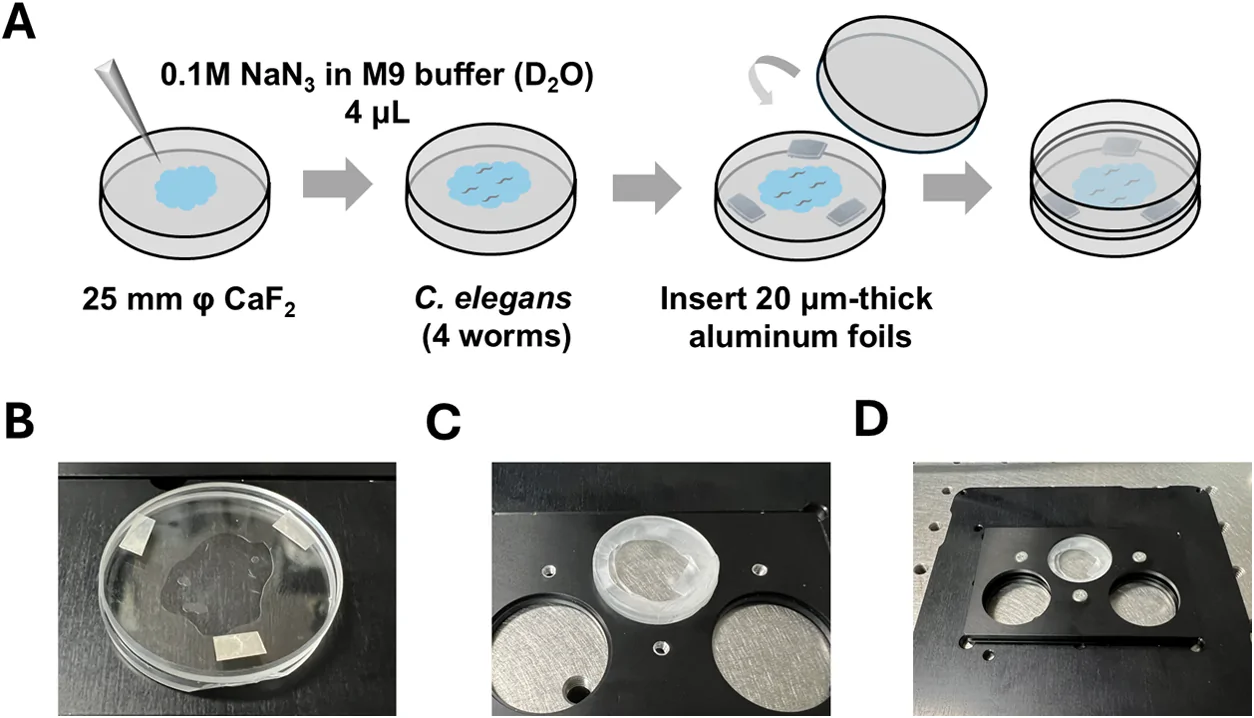
Sample preparation procedure for infrared imaging of *C. elegans*. (A) A 4 μL drop of M9 buffer (D_2_O) containing 0.1 M sodium azide (NaN_3_) was placed on a CaF_2_ window (25 mm in diameter and 2 mm thick), and four worms were transferred into the solution. (B) Optical image of the sample, showing worms immersed in D_2_O. (C) The sample chamber was sealed with Parafilm to prevent dehydration. (D) The sample was mounted on a metal holder with aluminum spacers (20 μm thickness) to maintain a constant thickness during measurement.

### 2.4 Infrared Microspectroscopy

Measurements were performed using a Vertex 80 spectrometer coupled to a Hyperion 3000 microscope (Bruker) equipped with a Schwarzschild-type 15× IR objective lens and a 64 × 64 focal plane array (FPA) detector. The FPA detector was cooled by filling the liquid-nitrogen reservoir in two steps. An initial filling was used to cool the reservoir, followed by a short equilibration period before the final filling to ensure complete cooling of the detector. Then, the instrument and computer were powered on. The FPA detector was activated, and OPUS software was launched. Samples were mounted on the stage, focused in transmission mode, and images were acquired in either Single Image or Large Area mode. Background spectra were recorded on a blank area in the CaF_2_ windows (64 scans, 8.0 cm^–1^ resolution). After refining the focus in VIS mode, the optical path was switched to IR mode and sample spectra were collected under optimized measuring conditions (64 scans, 8.0 cm^–1^ resolution). Infrared hyperspectral images were acquired over the spectral range of 3850–750 cm^–1^ with the zero-filling factor of 2. During FPA imaging, the microscope knife-edge aperture was fully opened. The field of view for each image was approximately 170 × 170 μm, corresponding to a pixel size of ∼2.7 μm. The effective spatial resolution was limited by diffraction of mid-IR light and was therefore on the order of several micrometers, depending on wavelength. No additional atmospheric correction (CO_2_ or H_2_O compensation) was applied, as background and sample spectra acquired under nitrogen-purged conditions effectively minimized atmospheric contributions.

### 2.5 Second-Derivative Analysis Using MATLAB

MATLAB (MathWorks) equipped with the Hyperspectral Imaging Library and Image Processing Toolbox was used for spectral processing. The infrared spectra were offset so that the absorbance was set to zero at 3700 cm^–1^, and a baseline slope correction was subsequently applied so that the absorbance was also close to zero at 1900 cm^–1^. Then, the spectra were smoothed using the Savitzky–Golay method (seven-point window, five iterations), followed by second-order differentiation. To quantify the relative contributions of α-helical and β-sheet structures in the Amide I band, the ratio was calculated using either absorbance or second-derivative values according to the expression, 1628 cm^–1^/1628 + 1658 cm^–1^. In this formulation, the value approaches 1 when β-sheet structures dominate, 0.5 when α-helix and β-sheet contributions are comparable, and 0 when α-helical structures predominate. Infrared images were subsequently constructed based on this ratio. When the second-derivative values became very small or negative, the calculated ratio yielded unrealistically large or small values. To avoid this, a threshold value (1.0 × 10^–4^) was applied, and values below this threshold were set to a constant of 1.0 × 10^–4^ for the second derivatives. As a result, in regions with low second-derivative signals, the calculated ratio converged to 0.5.

Additional custom scripts were developed for hyperspectral processing, visualization, and figure preparation. OPUS files were exported in ENVI format for MATLAB analysis, and spectra were also exported as DPT files and plotted in Igor Pro. All IR imaging data presented in this study were generated using MATLAB workflows.

### 2.6 Data Extraction and ROI (Region of Interest) Analysis

In the infrared images of *C. elegans*, four regions of interest (ROIs) were defined along the longitudinal axis. From anterior to posterior, the regions were designated as Head2, Head1, Middle, and Tail. Head2 corresponds to the anterior head region, including the pharyngeal muscle and neural tissues. Head1 represents the region posterior to the pharynx, where the body wall muscle region begins. Middle corresponds to the central body region, and Tail corresponds to the posterior end of the worm.

For each region, a total of 25 pixels (5 × 5; approximately 13.5 μm × 13.5 μm), including a representative pixel and its surrounding area, were selected for analysis. From each pixel, the absorbance values at 1628 cm^–1^ and 1658 cm^–1^ were extracted from the raw spectra, and the ratio 1628 cm^–1^/1628 + 1658 cm^–1^ was calculated. Similarly, second-derivative values at 1628 cm^–1^ and 1658 cm^–1^ were obtained, and the same ratio was calculated from the second-derivative spectra.

When calculating the ratio 1628 cm^–1^/1628 + 1658 cm^–1^ from second-derivative spectra, if the denominator (1628 + 1658 cm^–1^) was less than or equal to 1 × 10^–4^, it was set to 1 × 10^–4^. This threshold, approximately one-tenth of the typical second-derivative values, was introduced to avoid overestimation caused by extremely small denominators.

For each ROI, the mean value of the 25 pixels was calculated and used as the representative value for each body region of an individual worm. The same analysis was performed for eight worms (worm1–worm8). The resulting data set was organized with body regions (Head2, Head1, Middle, and Tail) as columns and individual worms as rows. Mean values and standard errors reported in Table 1 and Table S1 were calculated from these representative values.

**Table 1:** Mean Second-Derivative Values of Infrared Spectra with Standard Errors (SE) for the Head2, Head1, Middle, and Tail Regions, Obtained from Eight Worms

	Head2		Head1		Middle		Tail	
–2nd derivative value	Mean	SE	Mean	SE	Mean	SE	Mean	SE
1658 cm^–1^ (×10^–4^)	8.66	0.45	8.06	0.39	10.22	0.55	6.99	0.46
1628 cm^–1^ (×10^–4^)	5.36	0.39	7.24	0.47	8.02	0.56	5.37	0.29
1628 cm^–1^/1658 + 1628 cm^–1^	0.378	0.012	0.469	0.010	0.437	0.010	0.435	0.011

### 2.7 Statistical Analysis

Statistical analysis was performed using Igor Pro (WaveMetrics). Comparisons among Head2, Head1, Middle, and Tail were conducted using the representative values obtained for each region from individual worms.

For multiple comparisons among the four regions, Tukey’s test was performed using the *StatsTukeyTest* function in Igor Pro. For example, absorbance values at 1628 cm^–1^ in the IR absorption spectra were analyzed using the following command

StatsTukeyTestAbs_′1628_Head1′,Abs_′1628_Head2′,Abs_′1628_Middle′,Abs_′1628_Tail′

This analysis provided the mean difference between each pair of regions, standard error, Tukey’s *q* value, critical *q* value, and *P* value. The *q* value represents the Studentized range statistic used in Tukey’s multiple comparison test, and the *P* value indicates the probability associated with each pairwise comparison. Differences were considered statistically significant based on *P* values (*, *P* < 0.05; **, *P* < 0.01; ***, *P* < 0.001).

For pairwise comparisons between two regions within the same individual (e.g., Head1 and Head2), a paired *t* test was performed using the *StatsTTest* function with the/PAIR option. For example, ratios calculated from the absorbance values (1628 cm^–1^/1628 + 1658 cm^–1^) were analyzed using

StatsTTest/PAIRAbs_Ratio_Head′1′,Abs_Ratio_Head′2′

The paired *t* test yielded the number of pairs, mean difference, standard error of the mean difference, *t* value, degrees of freedom, critical value, and *P* value. Statistical significance was defined based on *P* values (*, *P* < 0.05; **, *P* < 0.01; ***, *P* < 0.001).

### 2.8 Assessment of Postimaging Recovery and Viability of *C. elegans*

After completion of infrared imaging, worms were carefully recovered from the sample holder and transferred onto NGM plates seeded with *E. coli* OP50. Approximately 100 μL of M9 buffer (H_2_O) was gently applied to the worms on the CaF_2_ window to facilitate their transfer. The four worms used in each imaging experiment were placed on the same recovery plate. For independent imaging experiments, separate plates were used.

Worm recovery was assessed on the day following imaging. Individuals exhibiting active sinusoidal locomotion were considered to have recovered. Worms that showed no movement in response to gentle stimulation with a worm pick were classified as nonviable, whereas those that resumed movement upon stimulation were considered alive. Representative images were recorded for documentation.

Reproductive capability was evaluated by monitoring the recovery plates for several days after imaging. The presence of progeny was taken as evidence that the worms retained reproductive function following the imaging procedure.

The results and discussion for the viability of worms after infrared imaging are described in Section 3.6.

## 3 Results and Discussion

### 3.1 Preparation of live *C. elegans* Suitable for Infrared Imaging

In this study, we employed the mutant strain (tm4975) of *C. elegans*, which is insensitive to visible and ultraviolet illumination.^24,25^ This property minimizes behavioral responses induced by UV/visible-light illumination during optical observation, thereby reducing potential physiological perturbations prior to infrared imaging.

To immobilize worms during measurements, sodium azide (NaN_3_) was applied because of its rapid and reversible inhibitory effect on mitochondrial cytochrome oxidase.^1^ At an optimized concentration of 0.1 M, NaN_3_ induces near-immediate immobilization by suppressing ATP synthesis, while allowing recovery approximately 30 min after removal. Although NaN_3_ can be lethal at high doses or with prolonged exposure,^26^ short-term treatment causes minimal physiological or structural damage, making it suitable for live-imaging applications. Compared with other anesthetics such as levamisole or 1-phenoxy-2-propanol (1P2P), both of which require several hours for behavioral recovery,^27,28^ NaN_3_ provides a distinct advantage in maintaining worm viability and enabling repeated measurements.

A second requirement for infrared microspectroscopy is minimizing interference from water absorption. Liquid H_2_O exhibits strong O–H stretching (∼3400 cm^–1^) and bending absorption (∼1640 cm^–1^), which overlaps the lipid C–H stretching bands (∼2900 cm^–1^) and the protein Amide I band (∼1650 cm^–1^) and complicates spectral interpretation.^20,21^ To circumvent this, all aqueous components were replaced with D_2_O-based buffer whenever feasible. Because the O–D stretching and bending vibrations of D_2_O appear at much lower wavenumbers (∼2400 cm^–1^ and ∼1200 cm^–1^, respectively), spectral overlaps with the C–H stretching and Amide I regions are greatly reduced. This D_2_O substitution allowed clearer detection of lipid- and protein-derived absorption bands within the worms during IR imaging.

### 3.2 Infrared Imaging of Lipid, Protein, and NaN_3_ Distributions in Immobilized *C. elegans*

Using a 64 × 64 focal plane array (FPA) detector, 4096 individual infrared spectra corresponding to a 170 × 170 μm field of view were acquired simultaneously within approximately 30 s–10 min depending on the measurement conditions (main factors are number of scans and spectral resolution) as shown in Table S7. A full-body scan of a single worm required as little as ∼3.5 min under practical measurement conditions (16 scans, 8.0 cm^–1^ spectral resolution) that provided sufficient spectral resolution and signal-to-noise ratio for analysis (Figures S1–S3). The optimization of spectral resolution and scan number is described in the Supplementary Results and Discussion (Figures S1–S18 and Table S7). Each absorbance band in the spectrum represents the absorption intensity at a given wavenumber, which depends on both the concentration of the absorbing species and the effective optical path length. Because the thickness of the worm body varies along its length and its internal anatomy is tubular, the optical path differs between pixels, complicating direct intensity comparison. To minimize this variation, the worm was gently flattened between 20-μm aluminum spacers, which standardized the sample thickness and enabled quantitative comparison among regions (Figure 1A). To determine optimal acquisition parameters, we systematically compared imaging conditions using spectral resolutions of 2, 4, and 8 cm^–1^ combined with 16, 64, 128, and 256 scans. For each condition, we evaluated total acquisition time (Table S7), spectral quality, signal-to-noise ratio, and overall IR image clarity. Based on a balanced consideration of measurement time and data quality, 8 cm^–1^ spectral resolution with 64 scans was concluded to provide the most practical and reliable condition. Accordingly, all results discussed below were obtained under these optimized settings. Data acquired under alternative conditions are presented in the Supporting Figures (S1–S18) for comparison where appropriate.

The optical image (Figure 2A) clearly showed the overall morphology of the worm and allowed identification of internal structures such as the intestine and gonad. The image at 2924 cm^–1^ (Figure 2B), corresponding to CH_2_ stretching vibrations of lipids, showed the strongest absorption in the midbody, particularly within the intestinal region. In contrast, the head and gonadal regions exhibited weaker signals, indicating more limited lipid distribution. Previous studies have reported stronger lipid band intensities near the central region of the worm body.^10^ In the present study, infrared imaging of living *C. elegans* revealed a more clearly defined intestinal morphology associated with lipid-rich regions. Lipid droplets localized in the intestine have also been reported using coherent Raman techniques such as CARS and SRS microscopy.^11,12^ Although the spatial resolution of mid-IR imaging is lower than that of these Raman-based methods, the observed lipid distribution is consistent with those findings. Furthermore, our IR images agree with previous reports indicating that the intestine serves as the primary site of lipid storage in *C. elegans*.^29^

**Figure 2:**
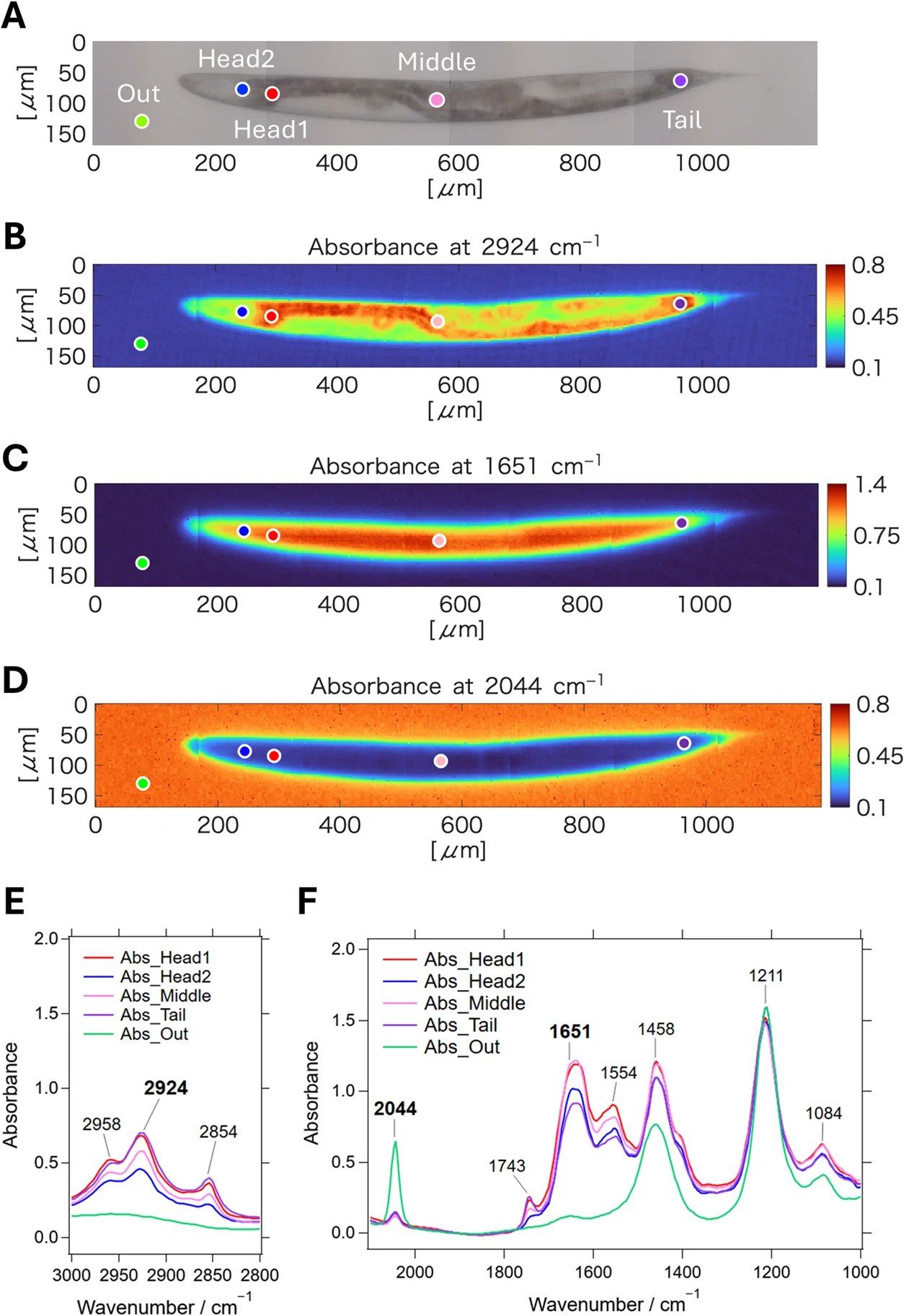
FT-IR imaging analysis of living *C. elegans* (A) Optical micrograph of a living worm showing its morphology and measurement region. (B–D) Infrared absorbance images. (B) 2924 cm^–1^: CH_2_ stretching vibration derived from lipids. (C) 1651 cm^–1^: Amide I vibration derived from proteins. (D) 2044 cm^–1^: Absorption derived from the azide group. The *X* and *Y* axes indicate the worm scale (μm). The color bar on the right represents infrared absorbance intensity. (E, F) Representative infrared absorbance spectra obtained from different regions. (E) 3000–2800 cm^–1^ (CH stretching region of lipids). (F) 2100–1000 cm^–1^ (region containing azide and amide bands). Spectra were collected from five positions (Head1 = red, Head2 = blue, Middle = pink, Tail = purple, Out = green).

The 1651 cm^–1^ image (Figure 2C), representing the Amide I band (mainly the C=O stretching vibration of peptide bonds in proteins), was distributed throughout the body. Absorption in the head region likely arises from pharyngeal muscles, whereas signals in the trunk and tail reflect body-wall muscles. The absorbance was highest in the central region and gradually decreased toward the head and tail according to thickness of the worm. The Amide I band will be analyzed in greater detail later using second-derivative processing.

The 2044 cm^–1^ image (Figure 2D) corresponds to the azide-ion (N_3_^–^) N≡N stretching band of NaN_3_. Moderate absorption near the cuticle suggests that NaN_3_ penetrates only superficially to induce immobilization, without diffusing deeply into internal tissues. A quantitative analysis based on the azide band will be described later.

The infrared spectra of the 3000–2800 cm^–1^ region (Figure 2E) showed three prominent peaks at 2958, 2924, and 2854 cm^–1^, assigned to the asymmetric CH_3_ stretching, asymmetric CH_2_ stretching, and symmetric CH_2_ stretching modes, respectively—typical signatures of saturated lipid chains.

The spectra in the 2100–1000 cm^–1^ region (Figure 2F) exhibited major peaks at 2044, 1743, 1651, 1554, 1458, 1211, and 1084 cm^–1^. The 2044 cm^–1^ band corresponds to the azide N≡N stretching vibration, and the 1743 cm^–1^ band reflects ester and phospholipid carbonyl C=O stretching vibration, indicative of membrane and storage lipids. The 1651 and 1554 cm^–1^ bands arise from protein Amide I (primarily α-helical C=O stretching) and Amide II (N–H bending/C–N stretching), respectively. The 1458 and 1211 cm^–1^ peaks originate from H–O–D and D–O–D bending vibrations of HOD and D_2_O, respectively, and the 1084 cm^–1^ peak corresponds to phosphate P=O stretching vibration. Excluding solvent-derived contributions, the spectra are dominated by lipid- and protein-related bands, each exhibiting distinct spatial distributions within the worm. In contrast, measurements performed in H_2_O resulted in a substantial reduction of spectral clarity, where the Amide I band was largely obscured and the lipid CH stretching bands showed significantly decreased definition due to strong water absorption (Figure S19).

In the present study, NaN_3_ was used for reversible immobilization of *C. elegans*. Azide ions exhibit a characteristic vibrational band at 2044 cm^–1^, enabling direct spectroscopic detection within the worm body. This distinct absorption allowed us to estimate the internal concentration of NaN_3_. In the external solution (0.1 M NaN_3_), the absorbance of the 2044 cm^–1^ band was 0.65. In contrast, the absorbance measured within the worm was approximately 0.12 in the midbody region and ∼0.15 in the head and tail regions. Assuming comparable optical path lengths and applying the Beer–Lambert law, these values correspond to estimated internal concentrations of approximately 22 mM in the midbody and 23 mM in the head and tail.

Even in the absence of a known reference concentration, the azide concentration can, in principle, be estimated from the absorbance of the N≡N stretching vibration, assuming that the optical path length is known. Using the molar absorption coefficient of the azide N≡N stretching vibration (∼400 M^–1^ cm^–1^)^13^ and an optical path length of 20 μm, concentrations estimated based on the Beer–Lambert law were approximately 0.81 M for the external solution, 0.15 M for the midbody region, and 0.19 M for the head and tail regions of the worm. These values are substantially higher than those estimated from the absorbance ratio using the external solution (0.1 M NaN_3_) as an internal calibration standard. Because the concentration of the external solution was precisely known under the same measurement conditions, the ratio-based estimates (22–23 mM) are considered to provide a more reliable approximation of the intracellular NaN_3_ concentration in the present study. The discrepancy between the two approaches likely arises from uncertainties in the effective optical path length, the literature molar absorption coefficient, and other experimental factors. Therefore, rigorous absolute quantification would require calibration using standards measured under conditions comparable to those employed for the biological samples.

Although careful consideration may be required for accurate concentration estimation, the ability to estimate molecular concentrations is a key advantage of infrared spectroscopy. In this study, it was demonstrated that, for compounds exhibiting distinct infrared absorption bands, local concentrations can be quantitatively estimated in living *C. elegans*. The detection limit depends on the signal-to-noise ratio of the absorption band; in the case of NaN_3_, the practical lower limit was approximately 0.01 in absorbance units, corresponding to an estimated concentration of ∼ 1.5 mM under the present experimental conditions.

Internal standard bands, such as the azide N_3_ stretching vibration, can be used to assess the uniformity of sample thickness. The use of spacers during sample preparation contributed to maintaining a uniform sample thickness across the imaging area. This was further supported by the nearly constant absorbance of the azide band in regions outside the worm, indicating that the liquid layer thickness was approximately uniform.

### 3.3 Second-Derivative Analysis of Infrared Imaging Data for Resolving Protein Secondary Structures

Infrared imaging data sets were processed and analyzed using MATLAB, which provides a robust computational environment for large-scale spectral handling and visualization. The Image Processing Toolbox and Hyperspectral Viewer were used for pixel-based image manipulation and three-dimensional spectral visualization. For each pixel, the corresponding IR spectrum was smoothed using the Savitzky–Golay filter, and the second derivative was subsequently calculated. The Savitzky–Golay method effectively suppresses noise while preserving spectral features, and derivative processing enhances subtle peak variations and facilitates the separation of overlapping bands originating from distinct protein secondary structures. The processed spectra were reconstructed into three-dimensional matrices and visualized as wavenumber-dependent second-derivative absorbance images.

The second-derivative image at 1658 cm^–1^ (Figure 3B) corresponds predominantly to α-helical structures, whereas the 1628 cm^–1^ image (Figure 3C) reflects contributions from β-sheet structures. In most regions of the worm, the α-helix–related signal was stronger than the β-sheet–related signal, consistent with the predominance of α-helical proteins in body-wall and pharyngeal muscles. Muscle tissue is mainly composed of myosin and actin, which are known to contain a high proportion of α-helical structures. Accordingly, much of the Amide I absorption at 1658 cm^–1^ is presumed to originate from muscle proteins.

**Figure 3:**
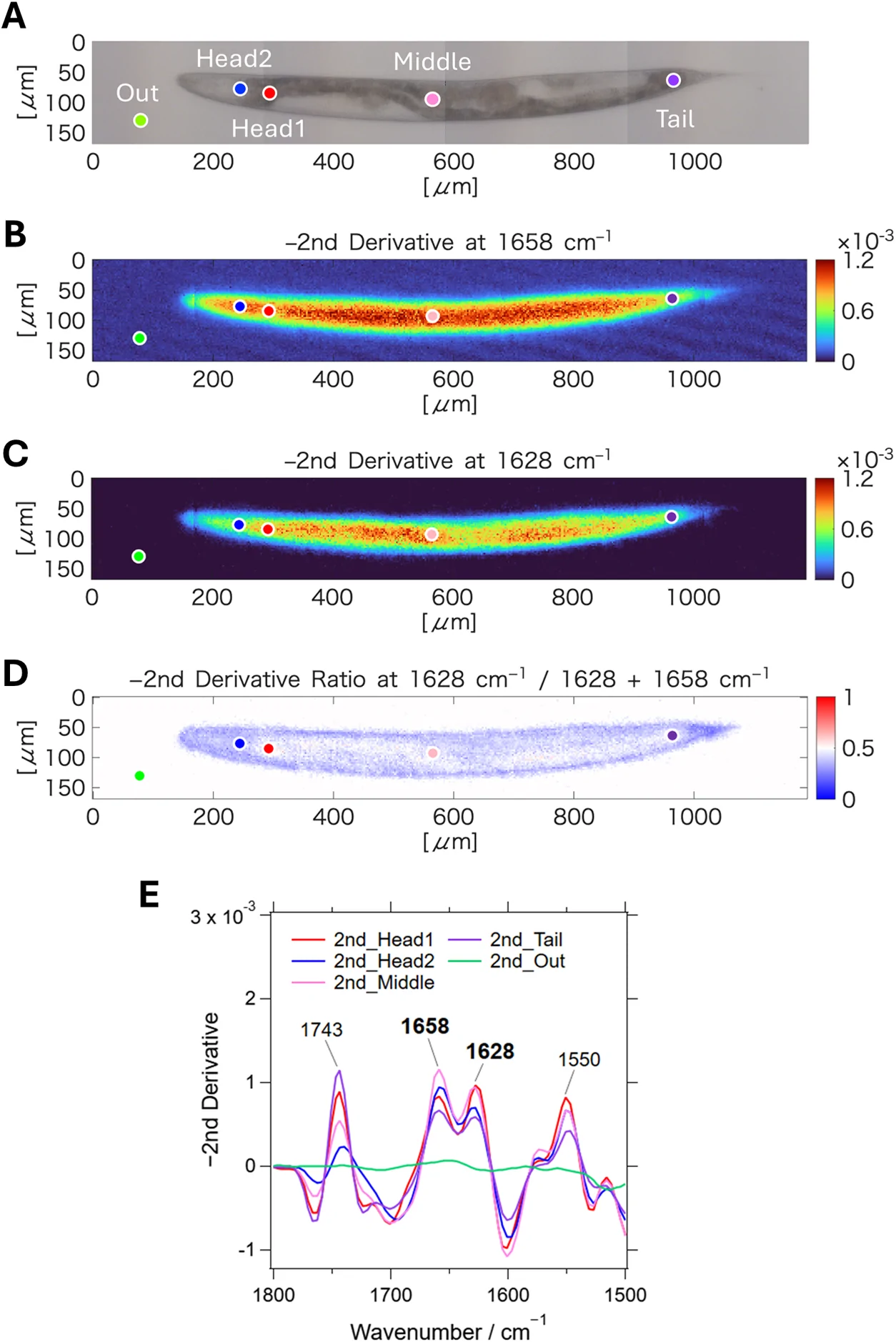
Distribution analysis of protein secondary structures by second-derivative processing (A) Optical micrograph of a living worm showing morphology and measurement region. (B, C) Second-derivative infrared absorbance images. (B) 1658 cm^–1^: α-helix structure within the Amide I bands. (C) 1628 cm^–1^: β-sheet structure within the Amide I bands. The right color bar indicates the second-derivative absorbance intensity. (D) Ratio image (1628 cm^–1^/1628 + 1658 cm^–1^) showing relative distribution of α-helix and β-sheet components. The *X* and *Y* axes indicate the worm scale (μm). (E) Representative infrared absorbance spectra (1800–1500 cm^–1^, including Amide bands) collected from five positions (Head1 = red, Head2 = blue, Middle = pink, Tail = purple, Out = green).

To evaluate spatial differences in protein conformation, a ratio image (1628 cm^–1^/1628 + 1658 cm^–1^) was generated (Figure 3D). Normalizing to the 1658 cm^–1^ and 1628 cm^–1^ bands compensates for regional variations in sample thickness and overall absorbance, enabling quantitative comparison of the relative β-sheet content. This analysis revealed pronounced inhomogeneity in secondary-structure composition along the worm body, suggesting the presence of site-specific conformational and environmental differences. Figure 3D shows that blue regions (<0.5) are predominant, indicating that β-sheet contributions are generally weaker than α-helical contributions. Notably, such regions are observed in the head and tail. The head region is discussed in further detail in the following section.

Because second-derivative analysis relies on precise measurement of the Amide I band, it cannot be reliably applied to spectra acquired in H_2_O, where this region is significantly obscured by strong water absorption (Figure S20).

It should be noted that the present measurements were performed in D_2_O rather than H_2_O. Although D_2_O is widely used in infrared spectroscopy to avoid spectral interference from water, isotope effects may alter enzyme kinetics, hydrogen-bonding networks, and protein conformational equilibria. Therefore, the measurements represent a subphysiological state rather than completely native physiological conditions. Although the Amide I band measured in D_2_O is sometimes referred to as the Amide I’ band to distinguish it from that measured in H_2_O,^20,21^ we use the term Amide I throughout this manuscript for simplicity. Since this vibration is dominated by the peptide C=O stretching mode, isotope-induced frequency shifts are relatively small and are not expected to affect the overall assignment of the major α-helical and β-sheet–related components.

Spectral differences among the head, trunk, and tail were also evident in the 1800–1500 cm^–1^ region (Figure 3E). The 1743 cm^–1^ lipid-associated carbonyl band exhibited minimal frequency shifts but clear intensity variations, consistent with regional lipid distribution. In contrast, differences in the Amide I (1658 and 1628 cm^–1^) and Amide II (1550 cm^–1^) bands reflected local variations in protein conformation, secondary-structure composition, and microenvironment.

To assess the effect of different immobilization methods on the results, additional infrared imaging experiments were conducted using levamisole. Under these conditions, immobilization of *C. elegans* using levamisole produced infrared images of comparable quality, and second-derivative analysis of the Amide I band yielded similar results (Figures S21 and S22). The concentration of levamisole (5 mM) was below the detection limit of the infrared imaging system, and unlike the azide (N_3_) band, no distinct spectral signal attributable to levamisole was observed (Figure S21E, F).

It should also be noted that the amplitudes of the absorbance and second-derivative spectra do not directly represent the content of individual secondary-structure components. In particular, second-derivative peak amplitudes are affected by both the intensity and the bandwidth of the corresponding absorbance bands. Therefore, the present interpretation assumes that the bandwidths of the major Amide I components do not vary substantially among the analyzed regions. Under this assumption, the second-derivative amplitudes provide an approximate measure of the relative contributions of the α-helical- and β-sheet-related components. More rigorous quantitative evaluation of secondary-structure content would require band fitting analysis and remain a subject for future investigation.

### 3.4 Statistical Validation of Spatial Variations in Protein Secondary Structure

The second-derivative infrared imaging shown in Figure 3 intuitively reveals regional differences in protein secondary structure. To confirm that these differences are reproducible and not due to individual biological variability, infrared imaging was performed on eight independent worms.

For each worm, the body was divided into four regions (Head2, Head1, Middle, and Tail), and second-derivative values at 1658 cm^–1^ and 1628 cm^–1^, as well as their ratio (1628 cm^–1^/1628 + 1658 cm^–1^), were calculated. The individual values for each worm are presented in Figure S23 and Table 1.

Mean values and standard errors were calculated, and statistical comparisons among regions were performed using Tukey’s test. The results showed that the 1658 cm^–1^ band was significantly higher in the Middle region than in the Head1 and Tail regions, reflecting differences in sample thickness (Figure 4A and Table 2). Similarly, the 1628 cm^–1^ band in the Middle region exhibited significantly higher values compared with the Head1 and Tail regions (Figure 4B and Table 3).

**Figure 4:**
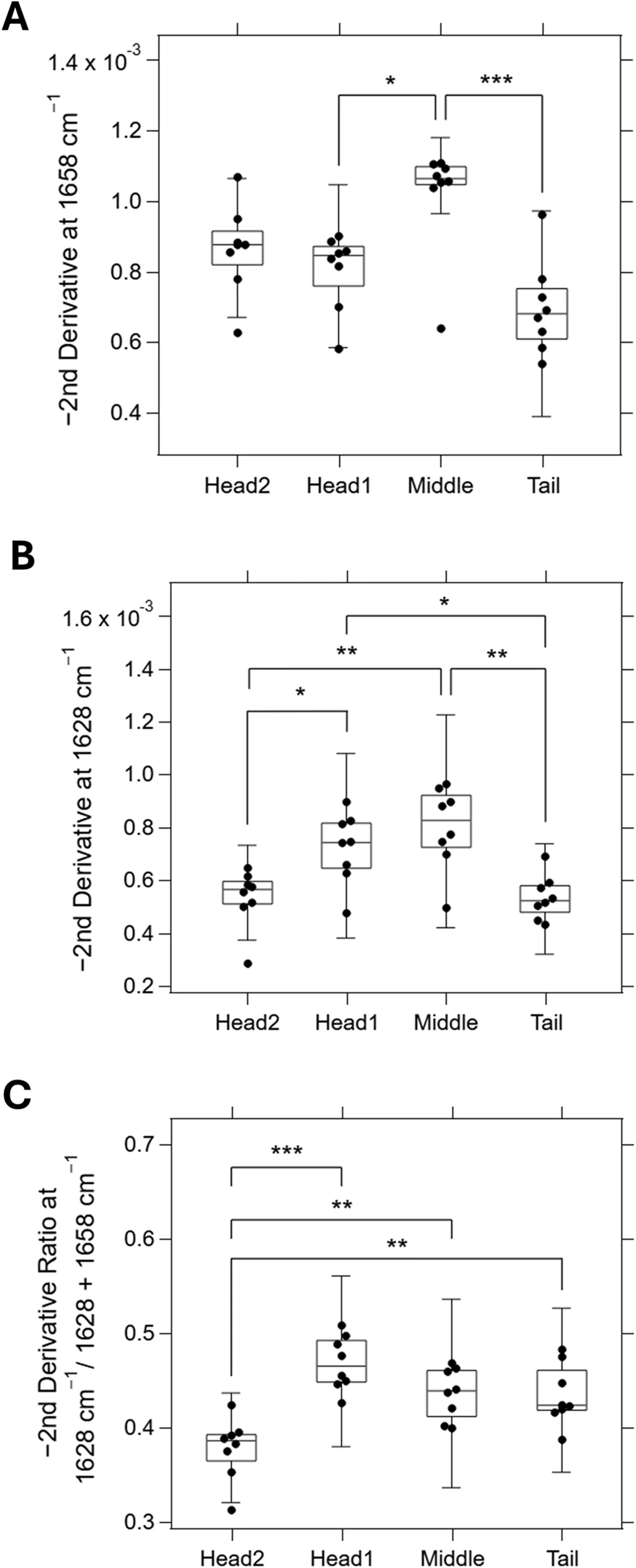
Box-and-whisker plots of second-derivative values at 1658 cm^–1^ (A) and 1628 cm^–1^ (B), and their ratio (1628 cm^–1^/1628 + 1658 cm^–1^) (C), obtained from eight worms. Each box represents the interquartile range (IQR), and whiskers extend to 1.5 × IQR. The median is indicated by a horizontal line within the box. Statistical significance determined by Tukey’s test is indicated as * (*P* < 0.05), ** (*P* < 0.01), and *** (*P* < 0.001). Detailed results of Tukey’s multiple comparison tests are provided in Tables 2–4

**Table 2:** Results of Tukey’s Multiple Comparison Test Based on the Second-Derivative Values of 1658 cm^**–1**^ Shown in Figure 4A and Table 1 ^a^

Pairwise comparison	Difference (×10^–4^)	*q* value	*P* value	Significance
Head2–Head1	0.60	1.28	0.801	n.s.
Middle–Head2	1.56	3.33	0.109	n.s.
Middle–Head1	2.16	4.62	0.0144	*
Head1–Tail	1.07	2.28	0.388	n.s.
Head2–Tail	1.66	3.57	0.0781	n.s.
Middle–Tail	3.22	6.90	0.000215	***

a Pairwise comparisons among the regions are listed, along with the mean difference, *q* value, *P* value, and significance level. The *q* value represents the Studentized range statistic used in Tukey’s multiple comparison test, and the *P* value indicates the probability associated with each pairwise comparison. Statistical significance is indicated as n.s. (not significant, *P* > 0.05), * (*P* < 0.05), and *** (*P* < 0.001).

**Table 3:** Results of Tukey’s Multiple Comparison Test Based on the Second-Derivative Values of 1628 cm^**–1**^ Shown in Figure 4B and Table 1 ^a^

Pairwise comparison	Difference (×10^–4^)	*q* value	*P* value	Significance
Head1–Head2	1.88	4.29	0.0250	*
Middle–Head2	2.66	6.07	0.00103	**
Tail–Head2	0.01	0.02	1.00	n.s.
Middle–Head1	0.78	1.78	0.596	n.s.
Head1–Tail	1.87	4.27	0.0259	*
Middle–Tail	2.65	6.05	0.00108	**

a Pairwise comparisons among the regions are listed, along with the mean difference, *q* value, *P* value, and significance level. The *q* value represents the Studentized range statistic used in Tukey’s multiple comparison test, and the *P* value indicates the probability associated with each pairwise comparison. Statistical significance is indicated as n.s. (not significant, *P* > 0.05), * (*P* < 0.05), and ** (*P* < 0.01).

Furthermore, the ratio 1628 cm^–1^/1628 + 1658 cm^–1^ in the Head2 region was significantly lower than in all other regions (Figure 4C and Table 4), indicating a reduced β-sheet contribution in this region. These results confirm that the spatial variations observed in the second-derivative images are statistically significant and reproducible.

**Table 4:** Results of Tukey’s Multiple Comparison Test Based on the Ratio of the Second-Derivative Values of 1628 cm^–1^/1628 + 1658 cm^–**1**^ Shown in Figure 4C and Table 1 ^a^

Pairwise comparison	Difference (×10^–4^)	*q* value	*P* value	Significance
Head1–Head2	0.090	8.45	0.000011	***
Middle–Head2	0.058	5.45	0.00330	**
Tail–Head2	0.057	5.29	0.00438	**
Head1–Middle	0.032	3.00	0.17030	n.s.
Head1–Tail	0.034	3.16	0.13890	n.s.
Middle–Tail	0.002	0.16	0.99952	n.s.

a Pairwise comparisons among the regions are listed, along with the mean difference, *q* value, *P* value, and significance level. The *q* value represents the Studentized range statistic used in Tukey’s multiple comparison test, and the *P* value indicates the probability associated with each pairwise comparison. Statistical significance is indicated as n.s. (not significant, *P* > 0.05), ** (*P* < 0.01), and *** (*P* < 0.001).

In contrast, statistical analysis of the absorbance values (shown in Figure S24 and Tables S1–S4) did not yield significant differences in the ratio values among regions (Figure S25C). This result highlights the effectiveness of second-derivative processing in enhancing the detection of subtle structural variations.

### 3.5 Localized Differences in α-Helix and β-Sheet Distributions Revealed by Magnified Analysis of the Head Region

To examine secondary-structure heterogeneity in more detail, the head region was magnified and analyzed using second-derivative imaging (Figure 5G–I). For comparison, infrared images without second-derivative processing are shown in Figure 5B–D. Figure 5A presents the corresponding visible image. In the 1658 cm^–1^ image, corresponding to α-helical structures (Figure 5G), strong signals were observed in the pharyngeal muscles and surrounding tissues. These muscles are rich in α-helical myosin and actin components, and the enhanced intensity is consistent with their well-organized, filamentous architecture.

**Figure 5:**
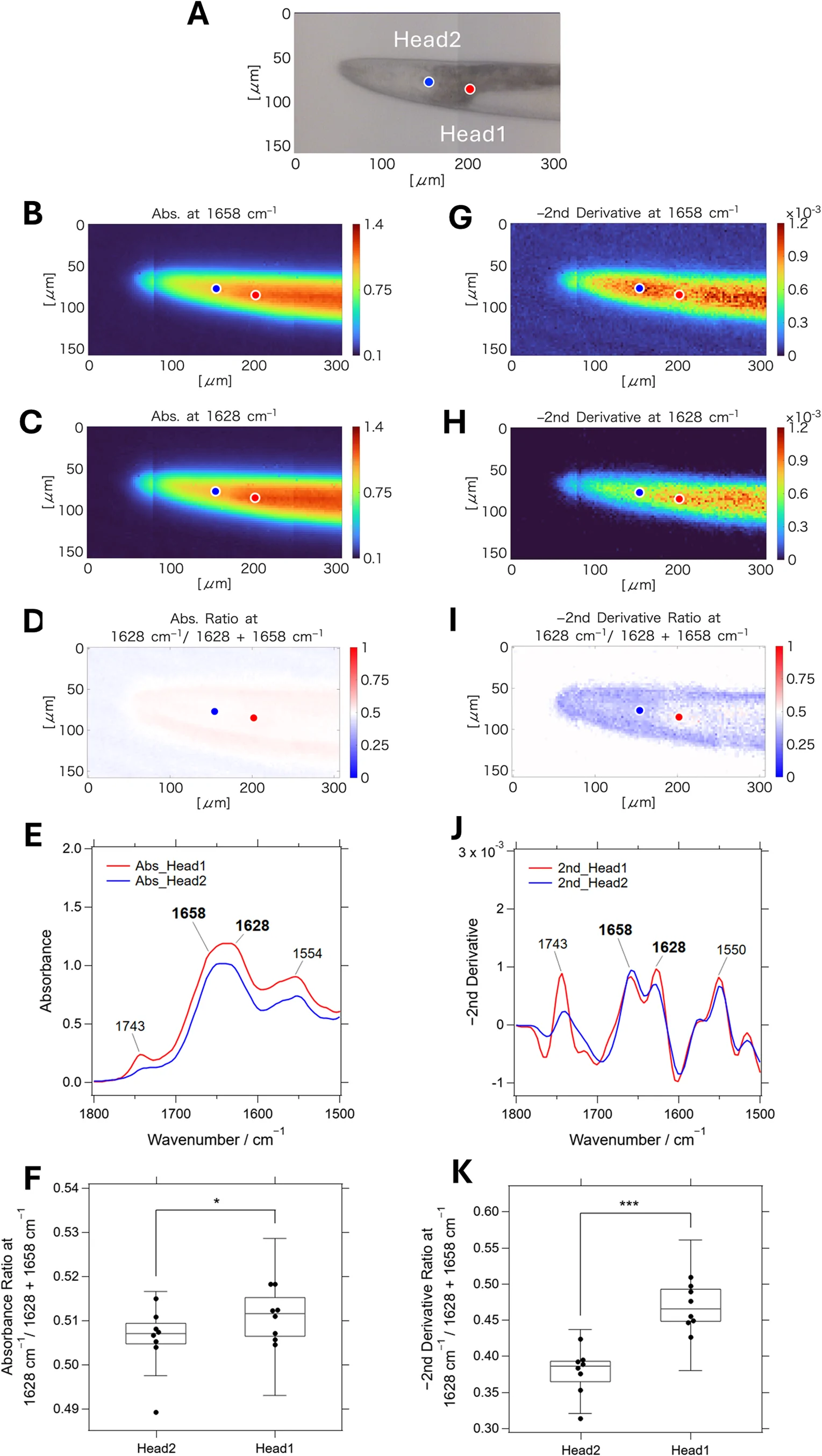
Comparison of pre- and postderivative IR images and spectra in the head region (A) Optical micrograph of the head region showing morphology and measurement area. (B, C) Prederivative images (B, 1658 cm^–1^ and C, 1628 cm^–1^). (D) Prederivative ratio image (1628 cm^–1^/1628 + 1658 cm^–1^). (G, H) Postderivative images (G, 1658 cm^–1^ and H, 1628 cm^–1^). (I) Postderivative ratio image (1628 cm^–1^/1628 + 1658 cm^–1^). The *X* and *Y* axes indicate scale in μm, and the color bar represents the intensity ratio. (E, J) Representative pre- (E) and postderivative (J) infrared absorbance spectra (1800–1500 cm^–1^) obtained from the two positions (Head1 = red, Head2 = blue). (F, K) Box plots of the ratio values (1628 cm^–1^/1628 + 1658 cm^–1^) for eight worms before (F) and after (K) second-derivative processing.

In contrast, the 1628 cm^–1^ image (Figure 5H), representing β-sheet–associated structures, displayed a markedly different spatial pattern. β-sheet–related signals were relatively weak in the core pharyngeal region and peripheral tissues, including the basal cuticle layer. The reduced β-sheet contribution in the pharyngeal region is more clearly visualized in the corresponding ratio image (Figure 5I) as the blue-colored area as described later. The latter region contains collagen proteins, which are known to be mostly composed of helical bundles.^30,31^ Therefore, reduction of β-sheet content may be partly explained by collagen distribution. We also consider that the higher proportion of α-helical structures of muscle proteins may account for the observed variation in the pharyngeal region.

The β-sheet ratio map (1628 cm^–1^/1628 + 1658 cm^–1^; Figure 5I) further emphasized these distinctions. The ratio was lower in the front pharyngeal region, indicating a predominance of α-helical proteins. These variations suggest that even within the relatively small head region, protein secondary-structure composition is not uniform but instead reflects the underlying tissue organization. The highly symmetric organization of the pharyngeal muscle has also been reported by second-harmonic generation (SHG) imaging.^12,32^ The high density of myosin and actin in this region suggests that the relative β-sheet content is reduced due to the predominance of α-helical muscle proteins.

To quantitatively evaluate this effect, statistical analysis was conducted using a paired *t* test. Without second-derivative processing, the comparison between Head1 and Head2 yielded a *P* value of 0.04 (Figure 5F and Table S5). In contrast, after second-derivative processing, the *P* value decreased markedly to 0.0004 (Figure 5K and Table S6), representing an approximately 100-fold increase in statistical significance. These results demonstrate that, even when derived from the same infrared imaging data set, second-derivative analysis significantly improves the ability to distinguish differences in protein secondary structure, both visually and statistically.

Taken together, the magnified second-derivative analysis demonstrates that IR imaging can resolve fine spatial variations in protein conformational composition within *C. elegans*. Because such contrast between α-helical and β-sheet structures was not observable without second-derivative processing (Figure 5B–D), the effectiveness of the second-derivative approach was clearly demonstrated for the first time. This capability enables high-spectral-resolution mapping of tissue-specific molecular architecture, providing a new approach for characterizing structural heterogeneity in living multicellular organisms.

It should be noted that second-derivative processing does not necessarily increase the statistical significance of every spectral parameter. Because the spectra were smoothed prior to derivative calculation, the observed differences are unlikely to arise primarily from increased spectral noise. Instead, second-derivative processing suppresses broad baseline variations, including residual contributions from H_2_O absorption in the Amide I region, especially at 1658 cm^–1^ (Table S2), thereby emphasizing changes associated with the Amide I band itself. Consequently, depending on the spectral parameter analyzed, statistical significance may either increase or decrease.

The ability of second-derivative infrared spectra to distinguish α-helical and β-sheet structures suggests broader applicability to diseases characterized by protein misfolding. Amyloidogenic disorders such as Alzheimer’s disease, driven by Aβ aggregation, or prion-related conditions including Creutzfeldt–Jakob disease, involve pronounced increases in β-sheet content during pathogenic conversion.^33^ The use of *C. elegans* genetically engineered to express Aβ protein has also been proposed as a platform for drug screening applications.^34^ The methodology demonstrated here may therefore provide a noninvasive approach for detecting and spatially mapping misfolded protein species *in vivo*. Extending IR imaging and derivative-based spectral decomposition to these systems could offer new opportunities for probing early aggregation events and tissue-specific structural changes associated with protein-misfolding diseases.

It should be noted that the present interpretation of α-helical and β-sheet contributions represents a simplified description of the Amide I band, which inherently contains overlapping components from multiple secondary structures. A more rigorous structural interpretation could be achieved by incorporating isotopic labeling (e.g., ^13^C or ^15^N) of proteins, lipids, and carbohydrates, which would provide additional spectral information for assigning individual biomolecular components. Complementary spectral analysis methods, such as Fourier self-deconvolution (FSD) or multivariate curve resolution–alternating least-squares (MCR-ALS), may further assist spectral interpretation and will be explored in future studies.

### 3.6 Assessment of *C. elegans* Viability after Infrared Imaging

During infrared imaging, worm locomotion was suppressed by NaN_3_-induced immobilization; therefore, postimaging recovery was explicitly evaluated to confirm survival. In each imaging experiment, four worms were mounted simultaneously. After measurements, all four individuals were carefully recovered and transferred to an NGM plate to assess restoration of movement.

Under acquisition conditions of 8 cm^–1^ spectral resolution, all four worms recovered normal locomotion in the first experiment (Figure S26A). A second independent imaging experiment under the same conditions again resulted in complete recovery of all four worms. In contrast, at 4 cm^–1^ resolution, three of four worms recovered in the first experiment (Figure S26B), and two of four recovered in the second experiment. At 2 cm^–1^ resolution, four worms recovered in the first trial (Figure S26C), whereas only one worm recovered in the second trial. Because higher wavenumber resolution requires longer acquisition times, the reduced recovery rate is likely attributable to prolonged exposure to NaN_3_ and D_2_O during measurement (Table S8).

The plates were re-examined several days later, confirming that the worm population had increased. Although no rigorous statistical analysis was performed, most worms recovered after infrared imaging retained their reproductive capability.

To our knowledge, this is the first study to explicitly verify postimaging survival following mid-IR hyperspectral imaging of living *C. elegans*. These results support the conclusion that the optimized acquisition conditions used in this study are minimally invasive and compatible with physiological recovery.

### 3.7 Implications of Mid-IR Imaging for Future Integration with Optogenetic Manipulation

Optogenetics has been extensively applied in *C. elegans* to control neuronal activity, muscle contraction, behavior, and programmed cell death.^35,36^ A variety of light-gated ion channels and microbial rhodopsins—including cation channelrhodopsins, anion channelrhodopsins (ACR1 and ACR2), the light-driven proton pump AR3, and GPCR-type rhodopsins such as LamPP and MosOpn3—have been successfully expressed in worms to modulate locomotion or induce apoptosis upon illumination.^25,35–37^ Because these optogenetic actuators are activated by visible light, their use often interferes with fluorescence microscopy or Raman-based imaging, both of which rely on visible or near-infrared excitation and may perturb optogenetic stimulation.

In contrast, mid-IR radiation interacts with fundamental vibrational modes and lies spectrally far from the excitation wavelengths typically used for optogenetics. Consequently, IR imaging provides a unique advantage: the imaging light does not activate rhodopsins, and optogenetic stimulation does not generate background signals in the IR spectral region. This spectral separation suggests that molecular IR imaging and optogenetic manipulation can be performed simultaneously without optical interference, enabling new experimental designs in which cellular or tissue-level responses to controlled neural or metabolic activation can be monitored in a living nematode.

The methodological framework established in this study opens the possibility of combining visible-light optogenetic stimulation with label-free IR chemical imaging to probe changes in protein conformation, lipid composition, or metabolite distribution during specific behavioral or physiological states. Furthermore, the introduction of IR-responsive chemical probes—such as azide (N ≡ N), cyano (C≡N), and deuterated (C–D) groups—could enable site-specific tracking of metabolic flux or protein-label dynamics using narrow, background-free vibrational markers. If the molar absorption coefficient of an IR probe is known, quantitative analysis should in principle be feasible, although the accuracy may depend on the concentration range. Such an integrated approach may provide powerful tools for dissecting the molecular mechanisms underlying behavior, energy metabolism, and cellular signaling in *C. elegans*.

## 4 Conclusions

In this study, we established an infrared imaging approach for intact *C. elegans* by combining reversible NaN_3_ immobilization, D_2_O-based buffer conditions, and controlled sample thickness using microspacers. This methodology enabled high-quality acquisition of hyperspectral data from individual worms within minutes. Second-derivative processing of pixel-resolved spectra allowed the separation of α-helix and β-sheet–related components within the Amide I band and revealed distinct spatial variations in protein secondary structures across tissues. In particular, magnified analysis of the head region suggested complementary distributions of α-helical muscle proteins and β-sheet–associated components.

These results demonstrate the feasibility of mid-IR imaging combined with derivative-based spectral analysis for visualizing molecular organization in living *C. elegans*. This approach provides a foundation for further validation and development, including studies under different biological conditions and in mutant strains. Ultimately, it may contribute to future investigations of structural dynamics, development, protein misfolding, and optogenetics in *C. elegans* and other small model organisms.

## Supplementary Material



## Notes

Infrared absorption spectra of *C. elegans*, their second derivative spectra, and the corresponding IR images with 2, 4, 8 cm^–1^ resolution obtained by 16–254 scans, which are discussed in a section of Supporting results and discussion (optimization of infrared imaging acquisition parameters). Results obtained from *C. elegans* in H_2_O and immobilized by levamisole for comparison. Line and box plots showing the variance of data and statistical analyses. Representative photos of *C. elegans* taken after IR measurements. Tables include parameters and results of statistical analyses, experimental conditions for infrared imaging, and a summary of postimaging survival rates of *C. elegans* (PDF)

## References

[1] Wood, W. B. The Nematode Caenorhabditis Elegans; Cold Spring Harbor Laboratory, 1988.

[2] Nigon V. M., Felix M. A. (2017). History of research
on *C. elegans* and other free-living
nematodes as model organisms. WormBook.

[3] Flavell S. W., Gordus A. (2022). Dynamic functional connectivity in
the static connectome
of *Caenorhabditis elegans*. Curr. Opin. Neurobiol..

[4] Horowitz L. B., Shaham S. (2024). Apoptotic and Nonapoptotic Cell Death
in *Caenorhabditis elegans* Development. Annu. Rev. Genet..

[5] Chalfie M., Tu Y., Euskirchen G., Ward W. W., Prasher D. C. (1994). Green fluorescent
protein as a marker for gene expression. Science.

[6] Hutter H. (2004). Five-colour
in vivo imaging of neurons in *Caenorhabditis elegans*. J. Microsc..

[7] Maddox A. S., Maddox P. S. (2012). High-resolution
imaging of cellular processes in *Caenorhabditis elegans*. Methods
Cell Biol..

[8] Haase K., Kroger-Lui N., Pucci A., Schonhals A., Petrich W. (2016). Real-time mid-infrared imaging of living microorganisms. J. Biophotonics.

[9] Sheng M., Gorzsas A., Tuck S. (2016). Fourier transform
infrared microspectroscopy
for the analysis of the biochemical composition of *C. elegans* worms. Worm.

[10] Bouyanfif A., Liyanage S., Hewitt J. E., Vanapalli S. A., Moustaid-Moussa N., Hequet E., Abidi N. (2017). FTIR imaging
detects
diet and genotype-dependent chemical composition changes in wild type
and mutant *C. elegans* strains. Analyst.

[11] Lee H. J., Cheng J. X. (2017). Imaging chemistry
inside living cells by stimulated
Raman scattering microscopy. Methods.

[12] Miyazaki S., Leproux P., Couderc V., Hayashi Y., Kano H. (2020). Multimodal
nonlinear optical imaging of *Caenorhabditis elegans* with multiplex coherent anti-Stokes Raman scattering, third-harmonic
generation, second-harmonic generation, and two-photon excitation
fluorescence. Appl. Phys. Express.

[13] Shi L., Liu X., Shi L., Stinson H. T., Rowlette J., Kahl L. J., Evans C. R., Zheng C., Dietrich L. E. P., Min W. (2020). Mid-infrared
metabolic imaging with vibrational probes. Nat.
Methods.

[14] Yin J., Zhang M., Tan Y., Guo Z., He H., Lan L., Cheng J. X. (2023). Video-rate mid-infrared photothermal imaging by single-pulse
photothermal detection per pixel. Sci. Adv..

[15] Golden J. W., Riddle D. L. (1984). The *Caenorhabditis elegans* dauer larva: developmental
effects of pheromone, food, and temperature. Dev. Biol..

[16] Karp X. (2018). Working with
dauer larvae. WormBook.

[17] Hedgecock E. M., Russell R. L. (1975). Normal and mutant
thermotaxis in the nematode *Caenorhabditis elegans*. Proc.
Natl. Acad. Sci. U.S.A.

[18] Mori I., Ohshima Y. (1995). Neural regulation of
thermotaxis in *Caenorhabditis elegans*. Nature.

[19] Mori Y., Ohta A., Kuhara A. (2025). Molecular,
neural, and tissue circuits
underlying physiological temperature responses in *Caenorhabditis
elegans*. Neurosci. Res..

[20] Barth A., Zscherp C. (2002). What vibrations tell
us about proteins. Q. Rev. Biophys..

[21] Barth A. (2007). Infrared spectroscopy
of proteins. Biochim. Biophys. Acta.

[22] Glasoe P. K., Long F. A. (1960). Use of Glass Electrodes to Measure Acidities in Deuterium
Oxide1,2. J. Phys. Chem. A.

[23] Altun, Z. F. ; Herndon, L. A. ; Wolkow, C. A. ; Crocker, C. ; Lints, R. ; Hall, D. H. WormAtlas 2002–2024, http://www.wormatlas.org.

[24] Gong J., Yuan Y., Ward A., Kang L., Zhang B., Wu Z., Peng J., Feng Z., Liu J., Xu X. Z. S. (2016). The *C. elegans* Taste
Receptor Homolog LITE-1 Is a Photoreceptor. Cell.

[25] Koyanagi M., Shen B., Nagata T., Sun L., Wada S., Kamimura S., Kage-Nakadai E., Terakita A. (2022). High-performance optical
control of GPCR signaling by bistable animal opsins MosOpn3 and LamPP
in a molecular property-dependent manner. Proc.
Natl. Acad. Sci. U.S.A..

[26] Massie M. R., Lapoczka E. M., Boggs K. D., Stine K. E., White G. E. (2003). Exposure
to the metabolic inhibitor sodium azide induces stress protein expression
and thermotolerance in the nematode *Caenorhabditis
elegans*. Cell Stress Chaperones.

[27] Manjarrez J. R., Mailler R. (2020). Stress and timing associated with *Caenorhabditis
elegans* immobilization methods. Heliyon.

[28] Davis A. N., Tanis J. E. (2022). Measuring *Caenorhabditis
elegans* Sensitivity to the Acetylcholine Receptor
Agonist Levamisole. J. Vis. Exp..

[29] Mak H. Y. (2012). Lipid droplets
as fat storage organelles in *Caenorhabditis elegans*: Thematic Review Series: Lipid Droplet Synthesis and Metabolism:
from Yeast to Man. J. Lipid Res..

[30] Payne K. J., Veis A. (1988). Fourier transform ir
spectroscopy of collagen and gelatin solutions:
Deconvolution of the amide I band for conformational studies. Biopolymers.

[31] Nashchekina Y. A., Starostina A. A., Trusova N. A., Sirotkina M. Y., Lihachev A. I., Nashchekin A. V. (2020). Molecular
and fibrillar structure
collagen analysis by FTIR spectroscopy. J. Phys.:
Conf. Ser..

[32] Campagnola P. J., Millard A. C., Terasaki M., Hoppe P. E., Malone C. J., Mohler W. A. (2002). Three-dimensional
high-resolution second-harmonic generation
imaging of endogenous structural proteins in biological tissues. Biophys. J..

[33] Hiramatsu H., Kitagawa T. (2005). FT-IR approaches on amyloid fibril
structure. Biochim. Biophys. Acta, Proteins
Proteomics.

[34] McColl G., Roberts B. R., Pukala T. L., Kenche V. B., Roberts C. M., Link C. D., Ryan T. M., Masters C. L., Barnham K. J., Bush A. I., Cherny R. A. (2012). Utility of an improved
model of amyloid-beta
(Aβ_1–42_) toxicity in *Caenorhabditis
elegans* for drug screening for Alzheimer’s
disease. Mol. Neurodegener..

[35] Tsukada Y., Mori I. (2021). Optogenetics in *Caenorhabditis elegans*. Adv.
Exp. Med. Biol..

[36] Nakao S., Kojima K., Sudo Y. (2022). Phototriggered Apoptotic
Cell Death
(PTA) Using the Light-Driven Outward Proton Pump Rhodopsin Archaerhodopsin-3. J. Am. Chem. Soc..

[37] Husson S. J., Gottschalk A., Leifer A. M. (2013). Optogenetic manipulation of neural
activity in *C. elegans*: from synapse
to circuits and behaviour. Biol. Cell.

