# Supplementary material for: Minimally Invasive Infrared Imaging of Caenorhabditis elegans Enabled by Reversible Chemical Immobilization and Second-Derivative Spectral Mapping
Source: ACS Omega. 2026 Aug 26;11(43):66163–75. doi: 10.1021/acsomega.6c03485 (PMC13737917; doi:10.1021/acsomega.6c03485)
Supplement: Supplementary file 1 [file ao6c03485_si_001.pdf]

Supporting Information

Minimally Invasive Infrared Imaging of *Caenorhabditis elegans* Enabled by Reversible Chemical Immobilization and Second-Derivative Spectral Mapping

Nodoka Wakita[1], *Akiko Ito*[1], *Ryu Sato*[2], *Akihisa Terakita*[2, 3], *Tatsuro Nishikino*[1, 4], *Mitsumasa Koyanagi*[2, 3], *and Yuji Furutani*[1, 4]*

[1]Department of Life Science and Applied Chemistry, Nagoya Institute of Technology, Showa-ku, Nagoya 466-8555, Japan.

[2]Department of Biology, Graduate School of Science, Osaka Metropolitan University, 3-3-138 Sugimoto, Sumiyoshi-ku, Osaka 558-8585, Japan.

[3]The OMU Advanced Research Institute for Natural Science and Technology, Osaka Metropolitan University, 3-3-138 Sugimoto, Sumiyoshi-ku, Osaka 558-8585, Japan.

[4]OptoBioTechnology Research Center, Nagoya Institute of Technology, Showa-ku, Nagoya 466-8555, Japan.

* Correspondence author's E-mail: furutani.yuji@nitech.ac.jp

Supplementary Results and Discussion

S1. Optimization of infrared imaging acquisition parameters

The quality of infrared images improves with increasing scan number; however, this improvement comes at the expense of longer acquisition time. Likewise, increasing the spectral resolution enhances band separation in the spectra and provides more detailed structural information. However, higher spectral resolution generally results in a reduced signal-to-noise (S/N) ratio. This decrease in S/N becomes more pronounced after second-derivative processing, which amplifies spectral noise. Consequently, increasing spectral resolution typically necessitates a higher number of scans to compensate for reduced S/N, further prolonging the total measurement time.

To obtain infrared images of *C. elegans* in the living state, it is essential to understand and balance these trade-offs. In this study, we systematically evaluated spectral resolutions of 2, 4, and 8 cm^{-1} combined with scan numbers ranging from 16 to 256 (Figures S1–S18).

At a spectral resolution of 8 cm^{-1}, high-quality infrared images were obtained even with 16 scans, and the corresponding second-derivative images were also satisfactory (Figure S1 and S4). Nevertheless, closer inspection revealed that 64 scans provided improved image clarity and superior spectral quality. Therefore, 8 cm^{-1} resolution with 64 scans was selected as the optimal acquisition condition for this study. To obtain a complete image of a single worm, seven adjacent fields of view were stitched together, requiring a total acquisition time of 6 min 47 s (Table S7). If shorter measurement time is prioritized, acquisition at 8 cm^{-1} with 16 scans reduces the total imaging time to approximately 3 min 30 s, which is roughly half of that required under the optimized condition.

At a spectral resolution of 4 cm^{-1}, the visual appearance of the infrared images was similar between 16 and 128 scans (Figure S7). However, in the infrared absorption spectra obtained with 16 scans, substantial noise was observed in the Amide I and II regions (Figures S8 and S9). Furthermore, the quality of the second-derivative infrared images was inferior to that obtained at 8 cm^{-1} resolution (Figure S10). The second-derivative spectra also exhibited greater variability, and no additional peaks attributable to the improved spectral resolution were detected (Figures S11 and S12).

Even at the same scan number, acquisition time at 4 cm^{-1} was slightly longer than at 8 cm^{-1} (Table S7). Moreover, doubling the spectral resolution resulted in approximately a twofold increase in data size. These factors represent disadvantages for rapid infrared imaging. Taken together, we concluded that a spectral resolution of 8 cm^{-1} is more suitable for measurements of *C. elegans* in the living state.

Finally, infrared imaging at a spectral resolution of 2 cm^{-1} was also evaluated. Compared with 4 cm^{-1}, further increases in acquisition time and data size were evident (Table S7). Nevertheless, a noteworthy observation was the appearance of an additional peak at 1641 cm^{-1} in the Amide I region after second-derivative processing (Figure S17). Because the measurements were performed in D_2O-based buffer, this band is unlikely to originate from the O–H bending vibration of water and is instead attributed to the Amide I mode. In this case, the 1641 cm^{-1} component may reflect contributions from

proteins in a random coil conformation. Thus, increasing the spectral resolution to 2 cm^{-1} may enable more detailed analysis of protein secondary structures.

However, to obtain a sufficient signal-to-noise ratio at this resolution, approximately 256 scans were required, resulting in a total imaging time of 70 min 44 s (Table S7). Maintaining worms in NaN_3 for such prolonged periods may impose physiological stress and potentially affect their normal biological state.[1, 2] Therefore, we concluded that a spectral resolution of 2 cm^{-1} is not suitable for infrared imaging of *C. elegans* in the living condition.

List of the contents in the supporting information

Figure S1: Comparison of infrared images acquired at 8 cm^{-1} spectral resolution with varying scan numbers (16, 32, and 64 scans) at 2924, 1651, and 2044 cm^{-1}.

Figure S2: Comparison of infrared absorption spectra from different positions of *C. elegans* (3000–2800 cm^{-1} and 2100–1000 cm^{-1}) acquired at 8 cm^{-1} spectral resolution with 16, 32, and 64 scans.

Figure S3: Comparison of infrared absorption spectra from different positions of *C. elegans* in the whole mid-infrared region acquired at 8 cm^{-1} spectral resolution with 16, 32, and 64 scans.

Figure S4: Comparison of 2nd derivative infrared images acquired at 8 cm^{-1} spectral resolution with varying scan numbers (16, 32, and 64 scans) at 1658, 1628 cm^{-1} and the ratio image at 1628 cm^{-1}/ 1628 + 1658 cm^{-1}.

Figure S5: Comparison of 2nd derivative infrared absorption spectra from different positions of *C. elegans* (1800–1500 cm^{-1}) acquired at 8 cm^{-1} spectral resolution with 16, 32, and 64 scans.

Figure S6: Comparison of 2nd derivative infrared absorption spectra from different positions of *C. elegans* in the whole mid-infrared region acquired at 8 cm^{-1} spectral resolution with 16, 32, and 64 scans.

Figure S7: Comparison of infrared images acquired at 4 cm^{-1} spectral resolution with varying scan numbers (16, 32, 64, and 128 scans) at 2924, 1651, and 2044 cm^{-1}.

Figure S8: Comparison of infrared absorption spectra from different positions of *C. elegans* (3000–2800 cm^{-1} and 2100–1000 cm^{-1}) acquired at 4 cm^{-1} spectral resolution with 16, 32, 64, and 128 scans.

Figure S9: Comparison of infrared absorption spectra from different positions of *C. elegans* in the whole mid-infrared region acquired at 4 cm^{-1} spectral resolution with 16, 32, 64, and 128 scans.

Figure S10: Comparison of 2nd derivative infrared images acquired at 4 cm^{-1} spectral resolution with varying scan numbers (16, 32, 64, and 128 scans) at 1658, 1628 cm^{-1} and the ratio image at 1628 cm^{-1}/ 1628 + 1658 cm^{-1}.

Figure S11: Comparison of 2nd derivative infrared absorption spectra from different positions of *C. elegans* (1800–1500 cm^{-1}) acquired at 4 cm^{-1} spectral resolution with 16, 32, 64, and 128 scans.

Figure S12: Comparison of 2nd derivative infrared absorption spectra from different positions of *C. elegans* in the whole mid-infrared region acquired at 4 cm^{-1} spectral resolution with 16, 32, 64, and 128 scans.

Figure S13: Comparison of infrared images acquired at 2 cm^{-1} spectral resolution with varying scan numbers (16, 64, and 256 scans) at 2924, 1651, and 2044 cm^{-1}.

Figure S14: Comparison of infrared absorption spectra from different positions of *C. elegans* (3000–2800 cm^{-1} and 2100–1000 cm^{-1}) acquired at 2 cm^{-1} spectral resolution with 16, 64, and 256 scans.

Figure S15: Comparison of infrared absorption spectra from different positions of *C. elegans* in the whole mid-infrared region acquired at 2 cm^{-1} spectral resolution with 16, 64, and 256 scans.

Figure S16: Comparison of 2nd derivative infrared images acquired at 2 cm^{-1} spectral resolution with varying scan numbers (16, 64, and 256 scans) at 1658, 1628 cm^{-1} and the ratio image at 1628 cm^{-1}/ 1628 + 1658 cm^{-1}.

Figure S17: Comparison of 2nd derivative infrared absorption spectra from different positions of *C. elegans* (1800–1500 cm^{-1}) acquired at 2 cm^{-1} spectral resolution with 16, 64, and 256 scans.

Figure S18: Comparison of 2nd derivative infrared absorption spectra from different positions of *C. elegans* in the whole mid-infrared region acquired at 2 cm^{-1} spectral resolution with 16, 64, and 256 scans.

Figure S19: FT-IR imaging analysis of living *C. elegans* in H_2O

Figure S20: Second-derivative processing of infrared images obtained in H_2O

Figure S21: FT-IR imaging analysis of living *C. elegans* using levamisole as an alternative immobilization method in D_2O

Figure S22: Second-derivative processing of infrared images of *C. elegans* immobilized by levamisole

Figure S23: Line plots of second-derivative values at 1658 cm^{-1} and 1628 cm^{-1}, and their ratio (1628 cm^{-1}/ 1628 + 1658 cm^{-1}) for eight individual worms.

Figure S24: Line plots of infrared absorption values at 1658 cm^{-1} and 1628 cm^{-1}, and their ratio (1628 cm^{-1}/ 1628 + 1658 cm^{-1}) for eight individual worms.

Figure S25: Box plots of the infrared absorption values for 1658 cm^{-1} and 1628 cm^{-1}, and their ratio values (1628 cm^{-1}/ 1628 + 1658 cm^{-1}) for eight worms.

Figure S26: Representative images confirming survival of *C. elegans* after infrared imaging at 8, 4, and 2 cm^{-1} resolution.

Table S1: Mean values of infrared absorbance with standard errors (SE) for the Head2, Head1, Middle, and Tail regions, obtained from eight worms.

Table S2: Results of Tukey's multiple comparison test based on the absorbance of 1658 cm^{-1} shown in Table S1.

Table S3: Results of Tukey's multiple comparison test based on the absorbance of 1628 cm^{-1} shown in Table S1.

Table S4: Results of Tukey's multiple comparison test based on the ratio of the absorbance of 1628 cm^{-1} / 1628 + 1658 cm^{-1} shown in Table S1.

Table S5: Paired t-test results for the ratio (1628 cm^{-1}/ 1628 + 1658 cm^{-1}) between Head1 and Head2 without second-derivative processing.

Table S6: Paired t-test results for the ratio (1628 cm^{-1}/ 1628 + 1658 cm^{-1}) between Head1 and Head2 after second-derivative processing.

Table S7: Experimental conditions for infrared imaging, including spectral resolution, number of scans, acquisition time, and data size.

Table S8: Summary of post-imaging survival rates of *C. elegans* under different spectral resolution conditions.

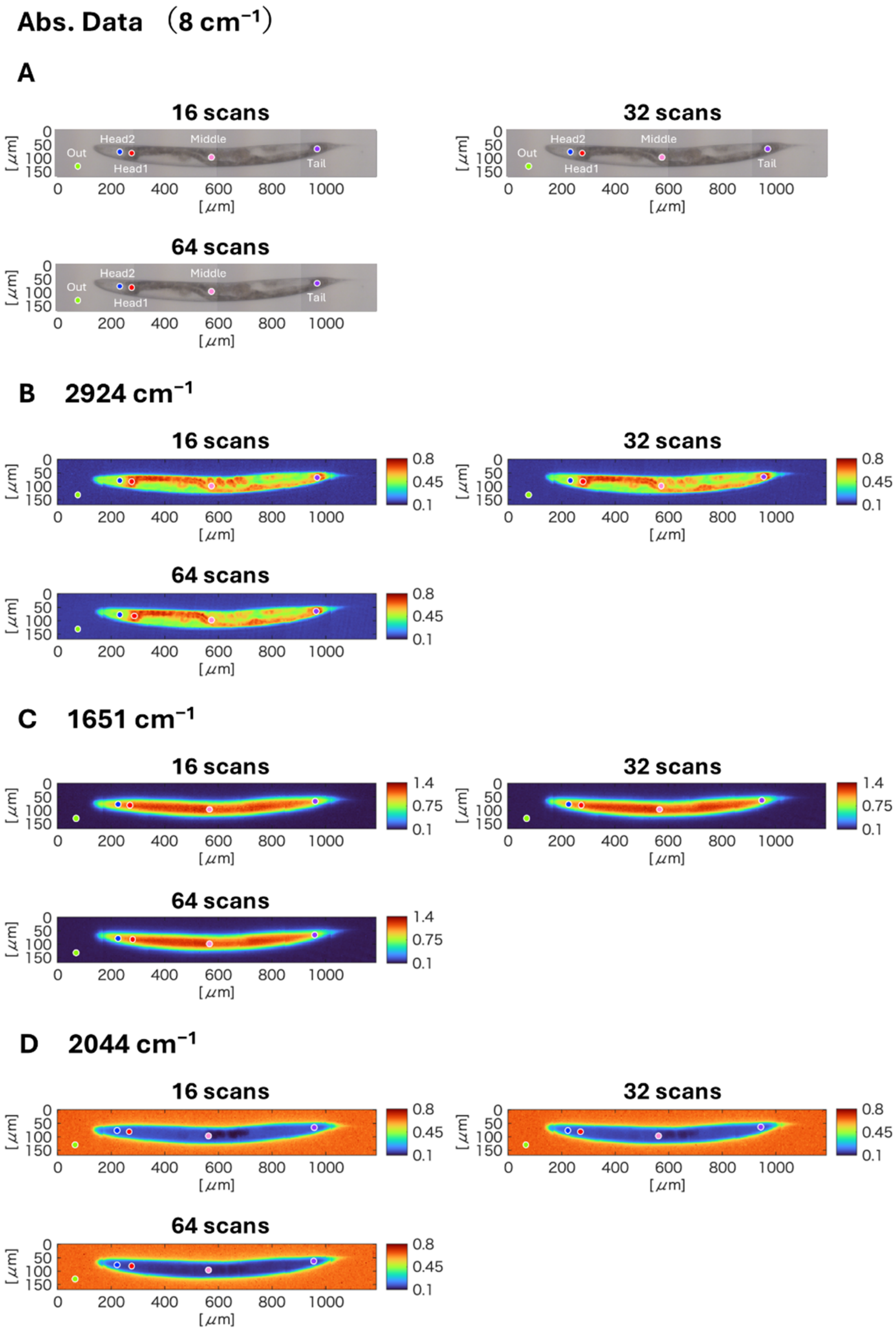

Figure S1. Comparison of infrared images acquired at 8 cm^{-1} spectral resolution with varying scan numbers (16, 32, and 64 scans) at 2924, 1651, and 2044 cm^{-1}. Infrared spectra obtained from the colored circles are shown in Figure S2 and S3 (Red circle: Head 1; blue circle: Head 2; pink circle: mid-body; purple circle: tail; green circle: external solution).

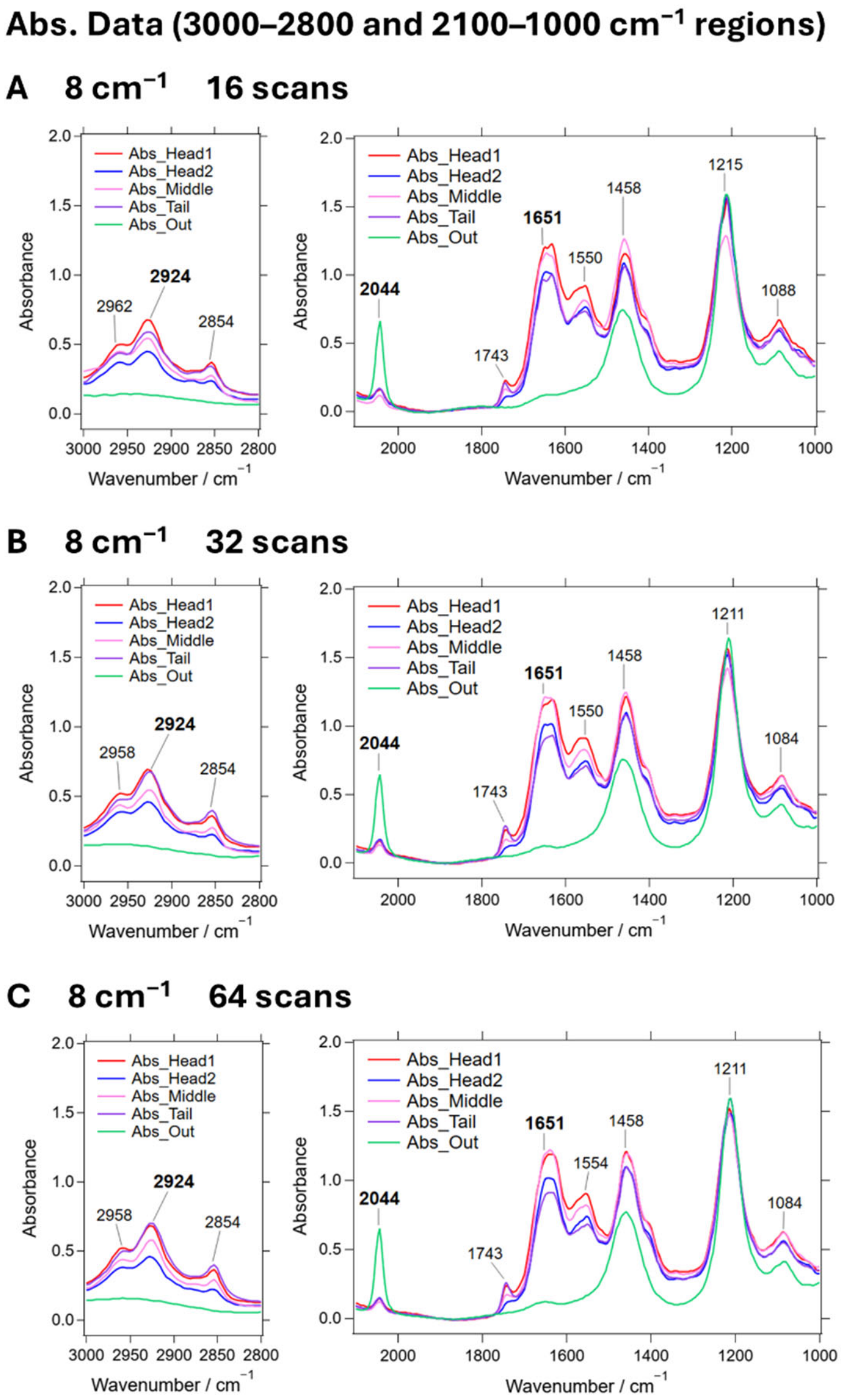

Figure S2. Comparison of infrared absorption spectra from different positions of *C. elegans* (3000–2800 cm^{-1} and 2100–1000 cm^{-1}) acquired at 8 cm^{-1} spectral resolution with 16, 32, and 64 scans. Infrared spectra were obtained from the following regions: red line (Head 1), blue line (Head 2), pink line (mid-body), purple line (tail), and green line (external solution). The exact sampling positions are shown as color-coded circles in Figure S1.

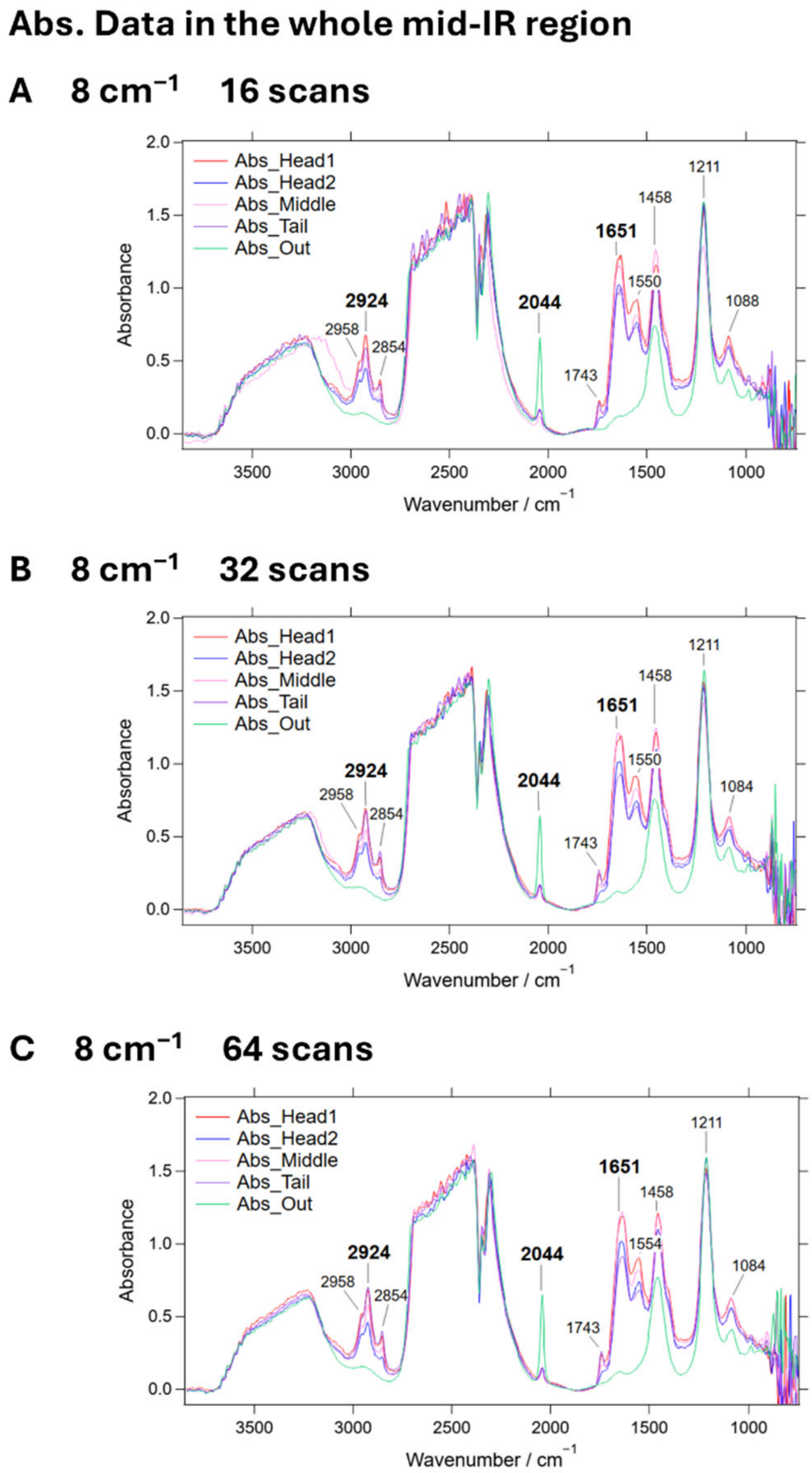

Figure S3. Comparison of infrared absorption spectra from different positions of *C. elegans* in the whole mid-infrared region acquired at 8 cm^{-1} spectral resolution with 16, 32, and 64 scans. Infrared spectra were obtained from the following regions: red line (Head 1), blue line (Head 2), pink line (mid-body), purple line (tail), and green line (external solution). The exact sampling positions are shown as color-coded circles in Figure S1.

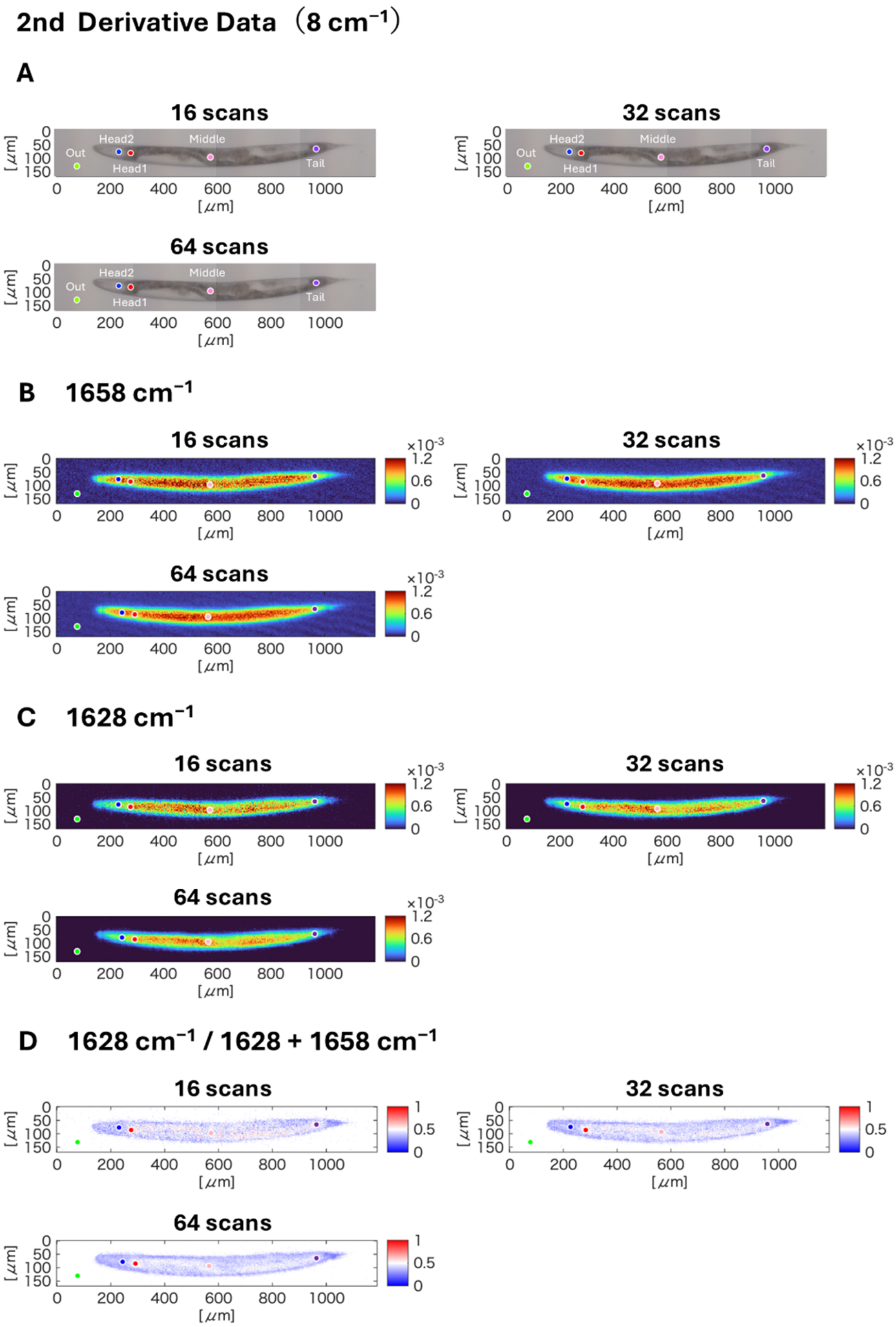

Figure S4. Comparison of 2nd derivative infrared images acquired at 8 cm^{-1} spectral resolution with varying scan numbers (16, 32, and 64 scans) at 1658, 1628 cm^{-1} and the ratio image at 1628 cm^{-1}/ 1628 + 1658 cm^{-1}. Second-derivative infrared spectra obtained from the colored circles are shown in Figure S5 and S6 (Red circle: Head 1; blue circle: Head 2; pink circle: mid-body; purple circle: tail; green circle: external solution).

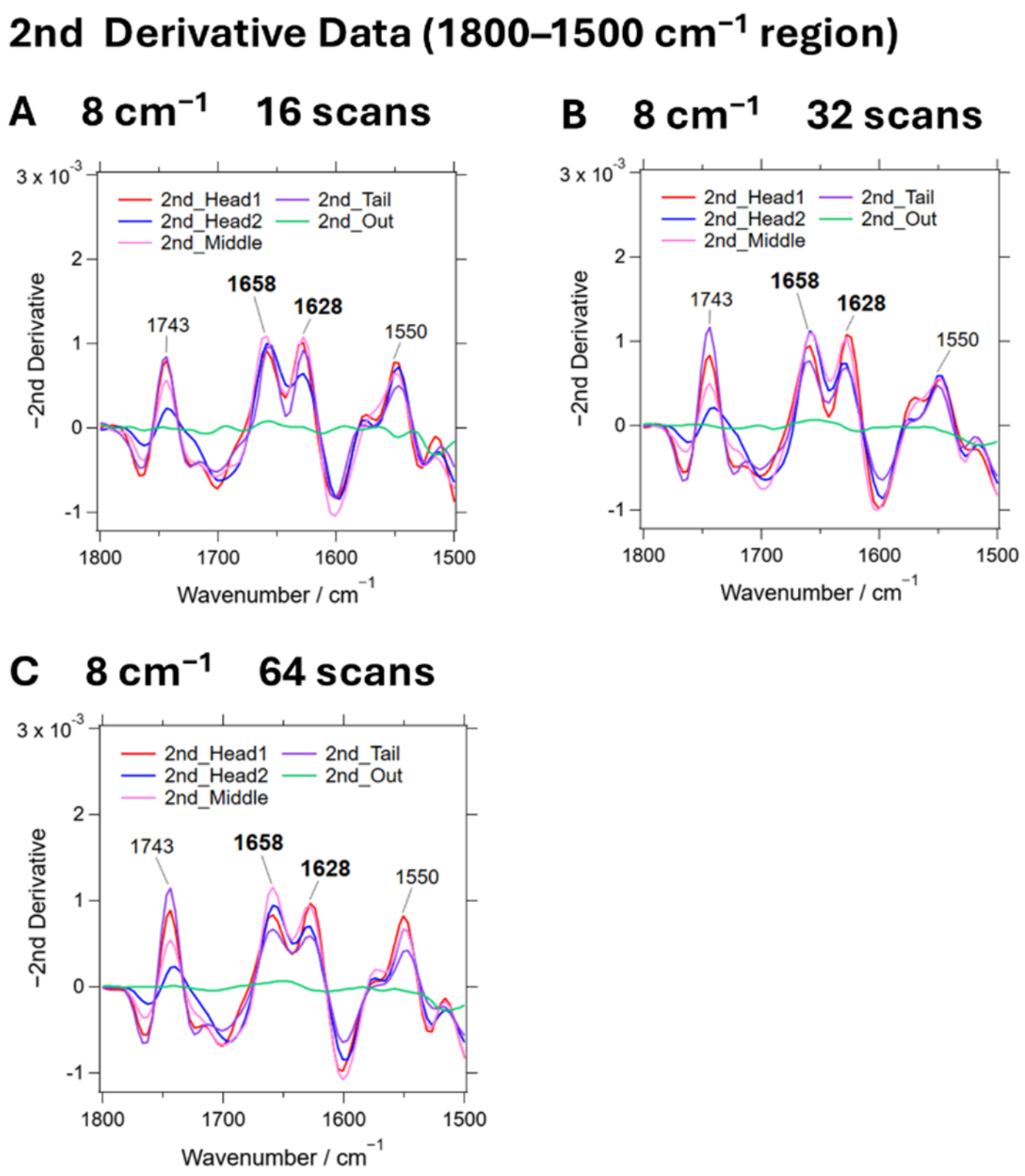

Figure S5. Comparison of 2nd derivative infrared absorption spectra from different positions of *C. elegans* (1800–1500 cm^{-1}) acquired at 8 cm^{-1} spectral resolution with 16, 32, and 64 scans. Second-derivative infrared spectra were obtained from the following regions: red line (Head 1), blue line (Head 2), pink line (mid-body), purple line (tail), and green line (external solution). The exact sampling positions are shown as color-coded circles in Figure S4.

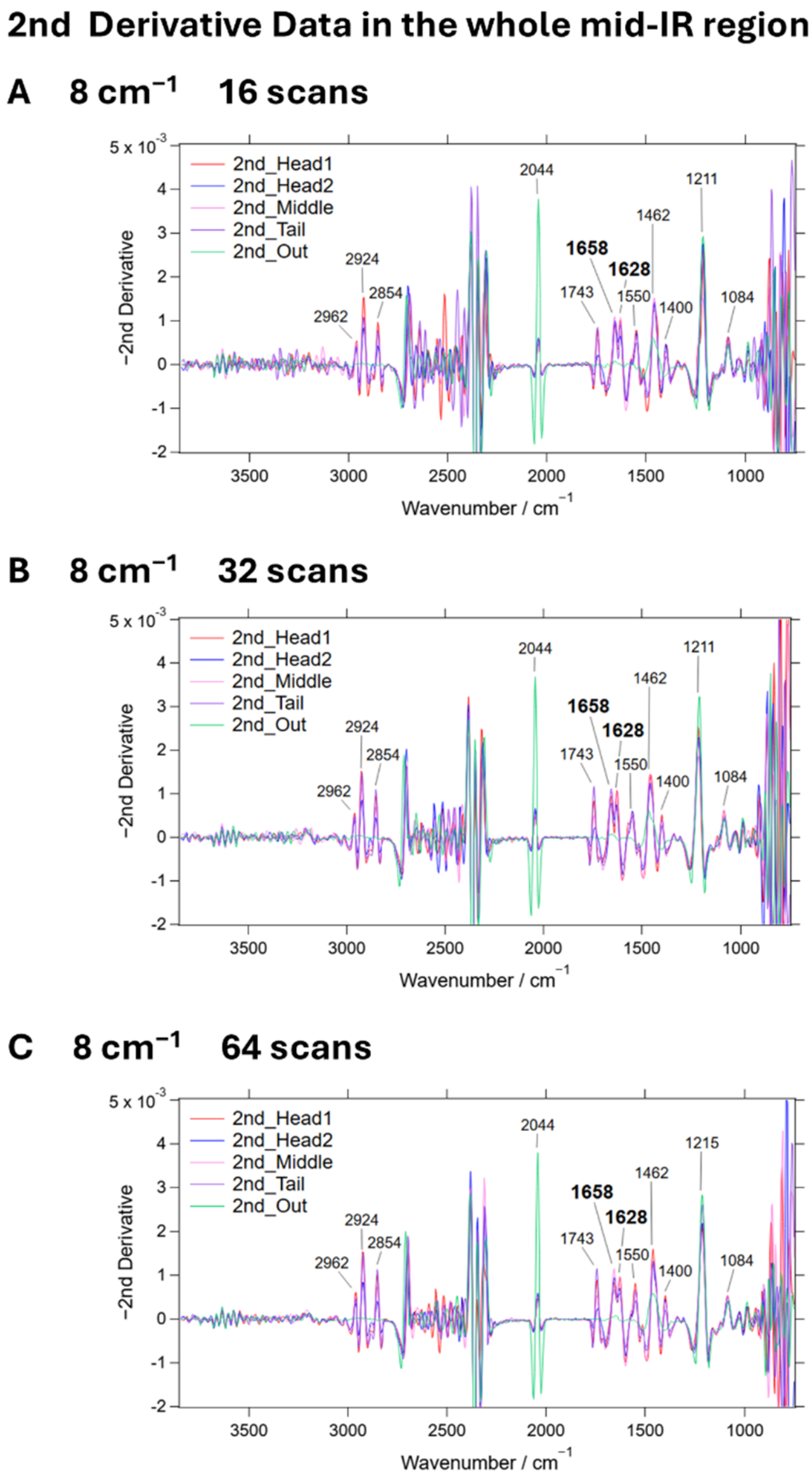

Figure S6. Comparison of 2nd derivative infrared absorption spectra from different positions of *C. elegans* in the whole mid-infrared region acquired at 8 cm^{-1} spectral resolution with 16, 32, and 64 scans. Second-derivative infrared spectra were obtained from the following regions: red line (Head 1), blue line (Head 2), pink line (mid-body), purple line (tail), and green line (external solution). The exact sampling positions are shown as color-coded circles in Figure S4.

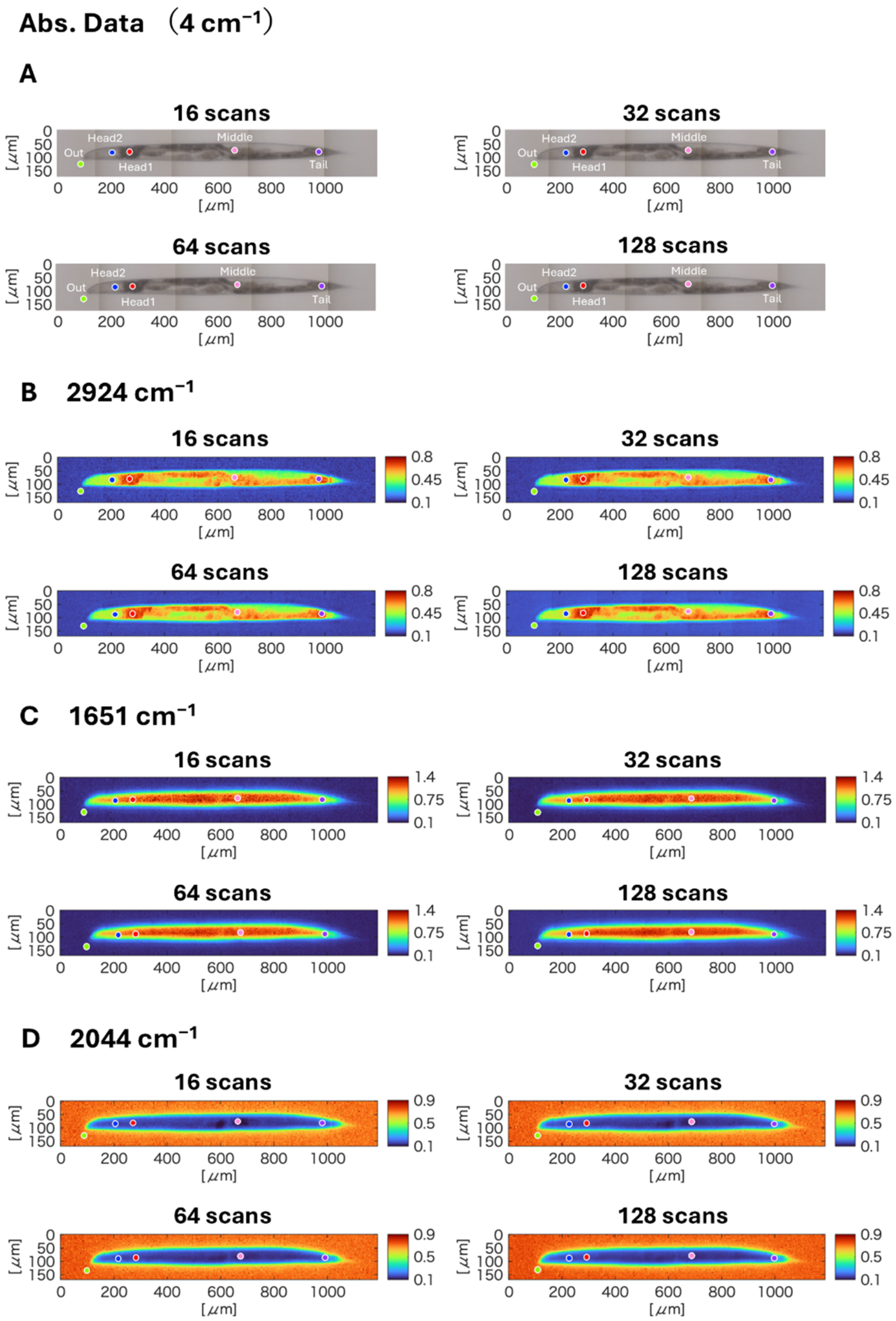

Figure S7. Comparison of infrared images acquired at 4 cm^{-1} spectral resolution with varying scan numbers (16, 32, 64, and 128 scans) at 2924, 1651, and 2044 cm^{-1}. Infrared spectra obtained from the colored circles are shown in Figure S8 and S9 (Red circle: Head 1; blue circle: Head 2; pink circle: mid-body; purple circle: tail; green circle: external solution).

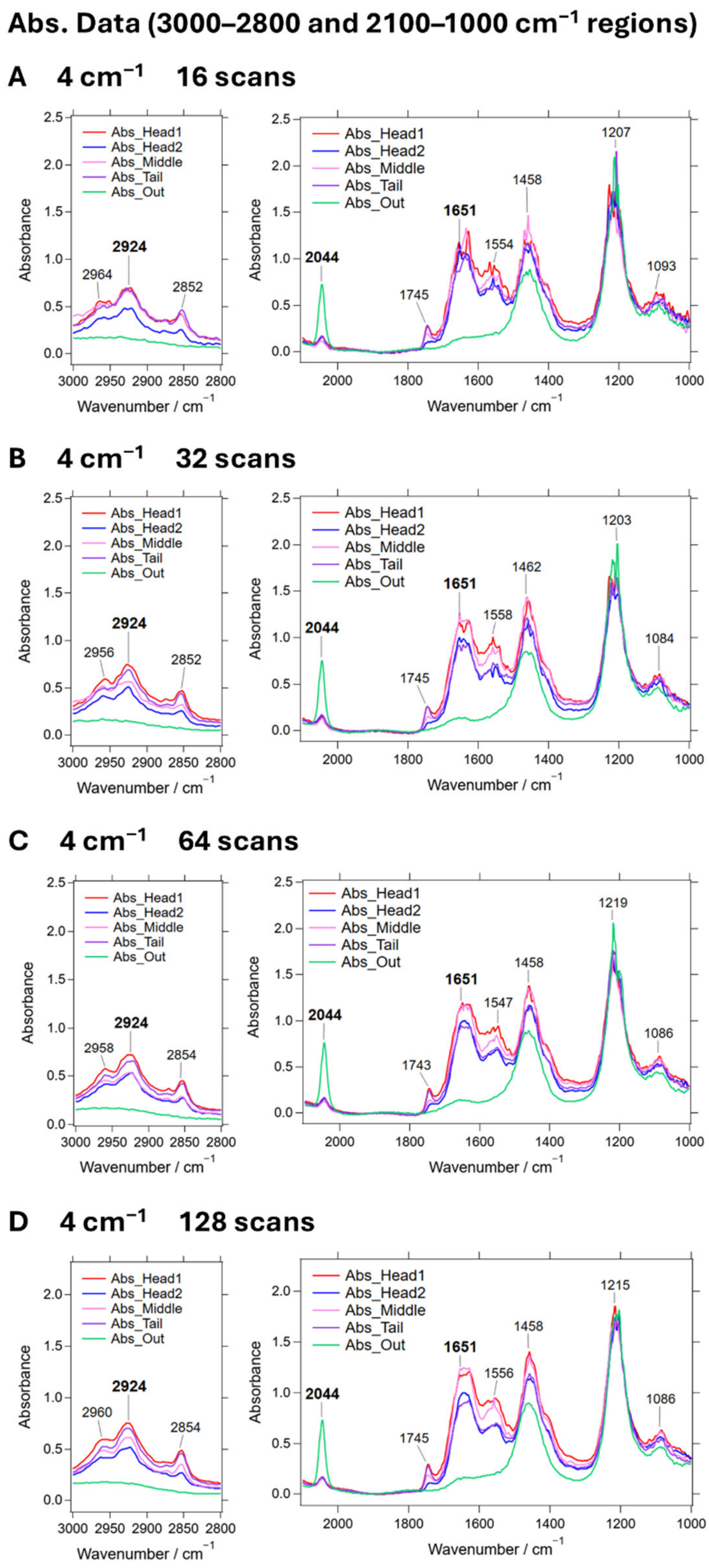

Figure S8. Comparison of infrared absorption spectra from different positions of *C. elegans* (3000–2800 cm^{-1} and 2100–1000 cm^{-1}) acquired at 4 cm^{-1} spectral resolution with 16, 32, 64, and 128 scans. Infrared spectra were obtained from the following regions: red line (Head 1), blue line (Head 2), pink line (mid-body), purple line (tail), and green line (external solution). The exact sampling positions are shown as color-coded circles in Figure S7.

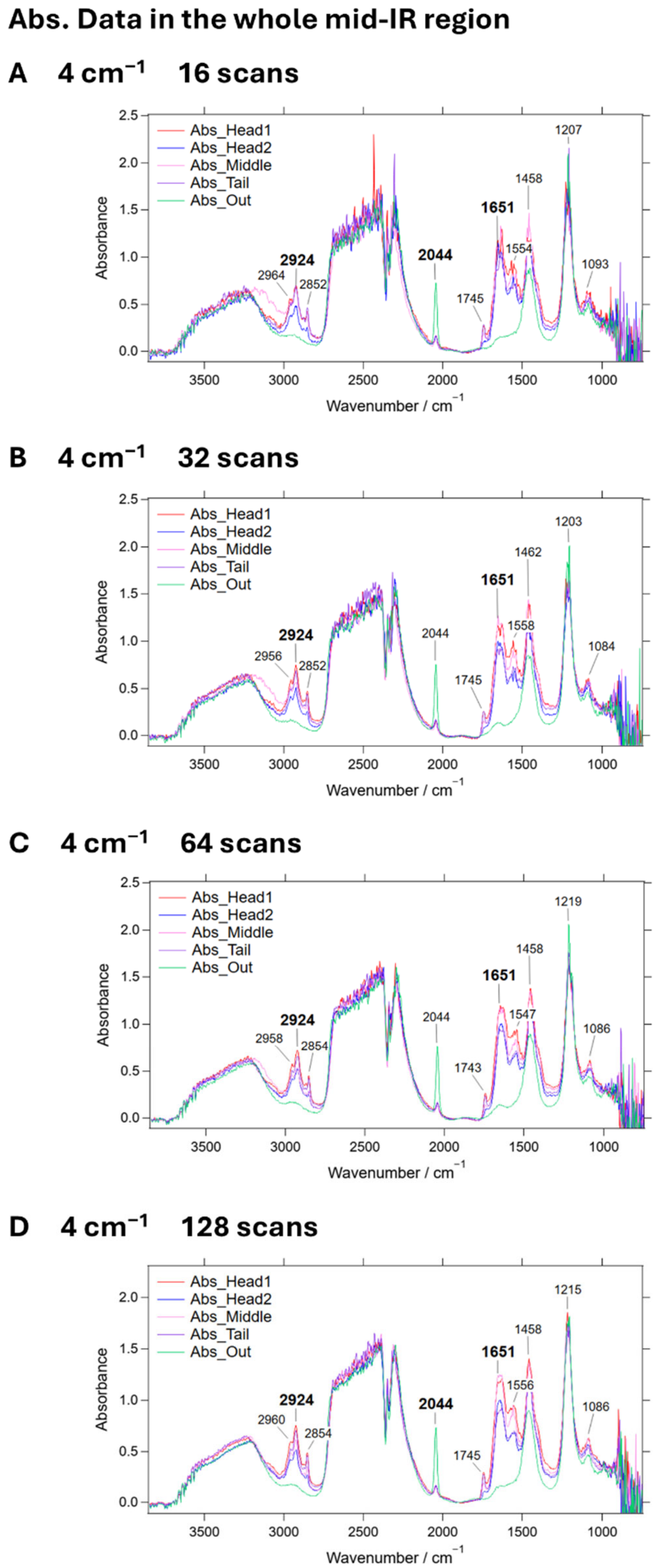

Figure S9. Comparison of infrared absorption spectra from different positions of *C. elegans* in the whole mid-infrared region acquired at 4 cm^{-1} spectral resolution with 16, 32, 64, and 128 scans. Infrared spectra were obtained from the following regions: red line (Head 1), blue line (Head 2), pink line (mid-body), purple line (tail), and green line (external solution). The exact sampling positions are shown as color-coded circles in Figure S7.

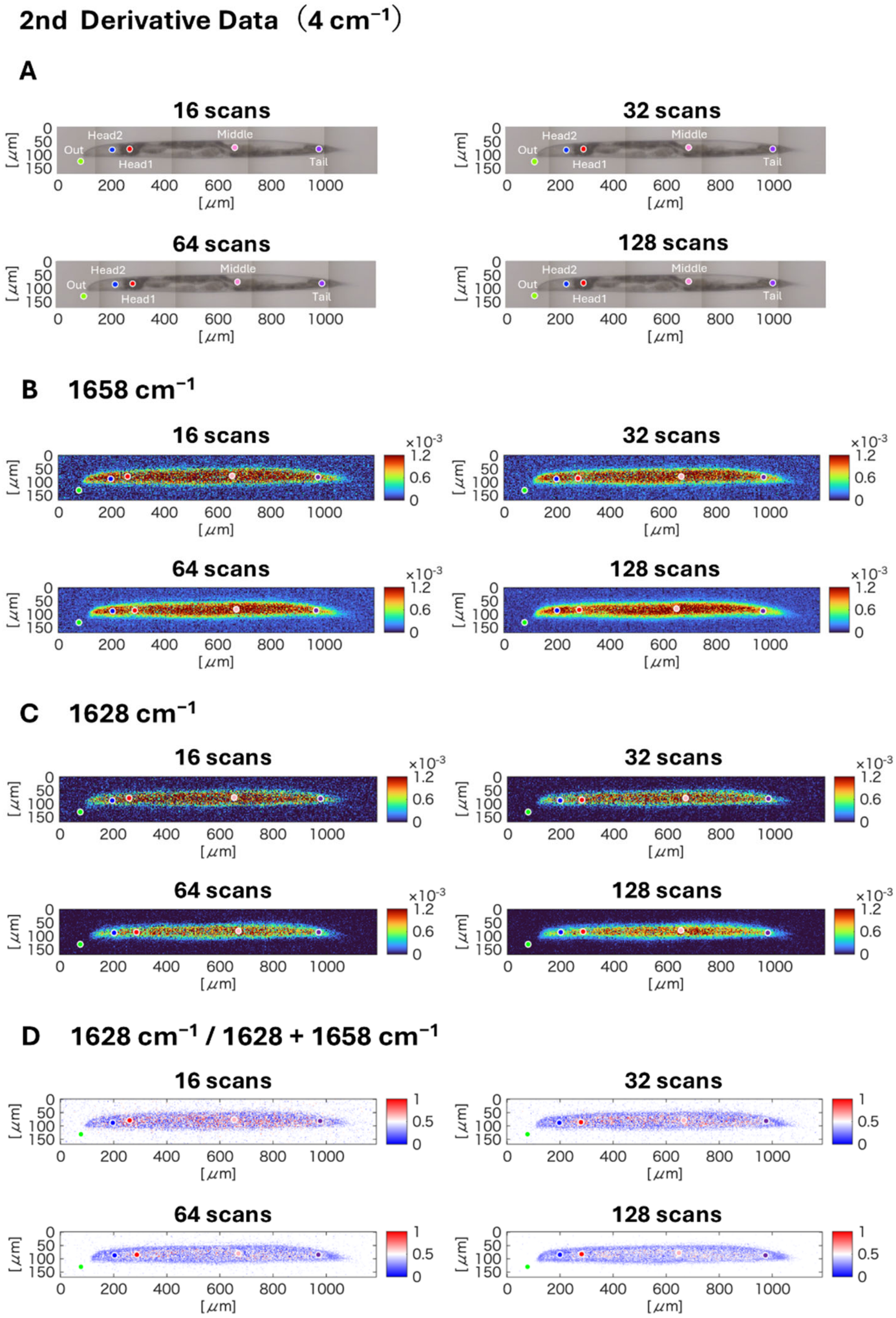

Figure S10. Comparison of 2nd derivative infrared images acquired at 4 cm^{-1} spectral resolution with varying scan numbers (16, 32, 64, and 128 scans) at 1658, 1628 cm^{-1} and the ratio image at 1628 cm^{-1}/ 1628 + 1658 cm^{-1}. Second-derivative infrared spectra obtained from the colored circles are shown in Figure S11 and S12 (Red circle: Head 1; blue circle: Head 2; pink circle: mid-body; purple circle: tail; green circle: external solution).

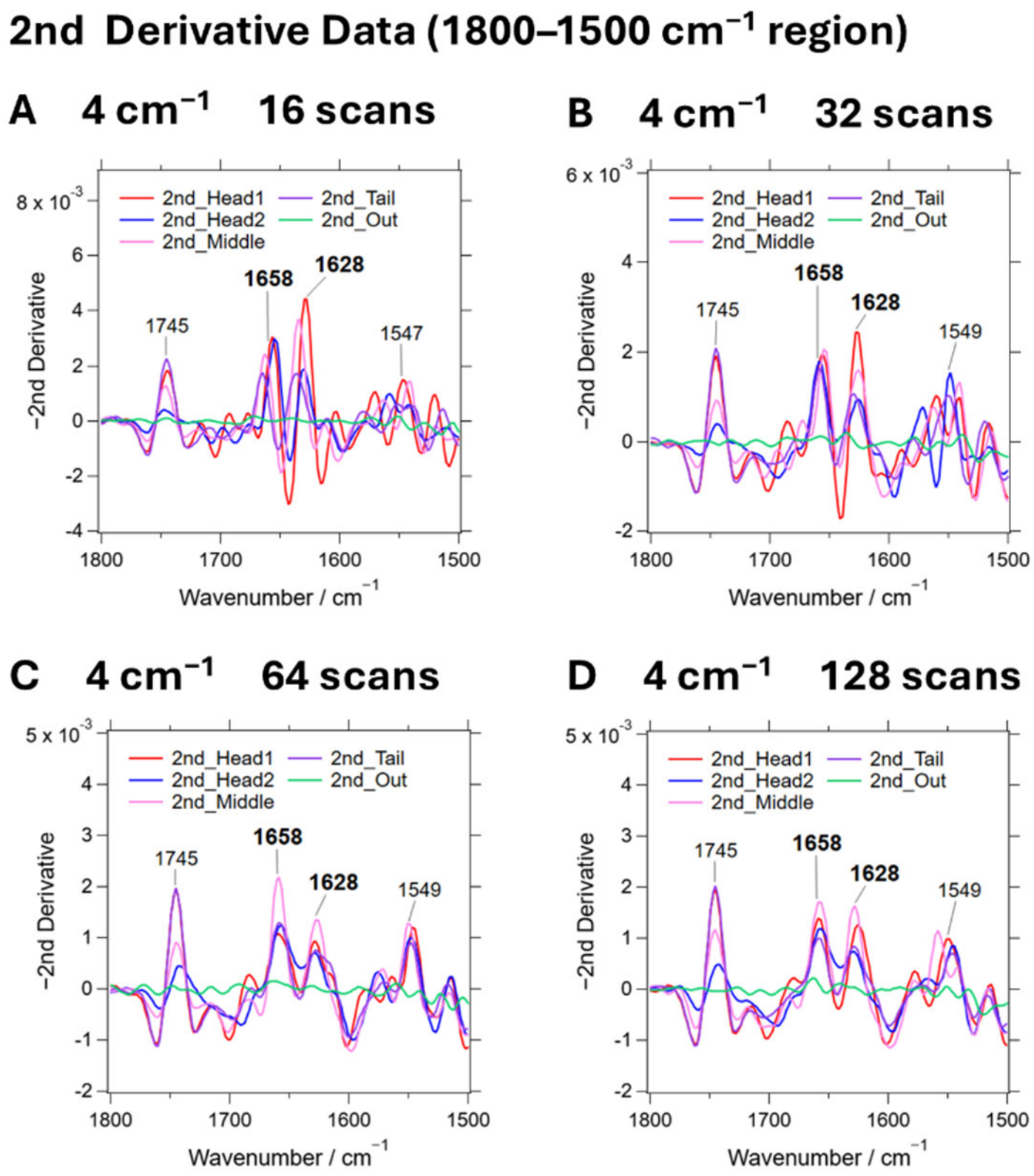

Figure S11. Comparison of 2nd derivative infrared absorption spectra from different positions of *C. elegans* (1800–1500 cm^{-1}) acquired at 4 cm^{-1} spectral resolution with 16, 32, 64, and 128 scans. Second-derivative infrared spectra were obtained from the following regions: red line (Head 1), blue line (Head 2), pink line (mid-body), purple line (tail), and green line (external solution). The exact sampling positions are shown as color-coded circles in Figure S10.

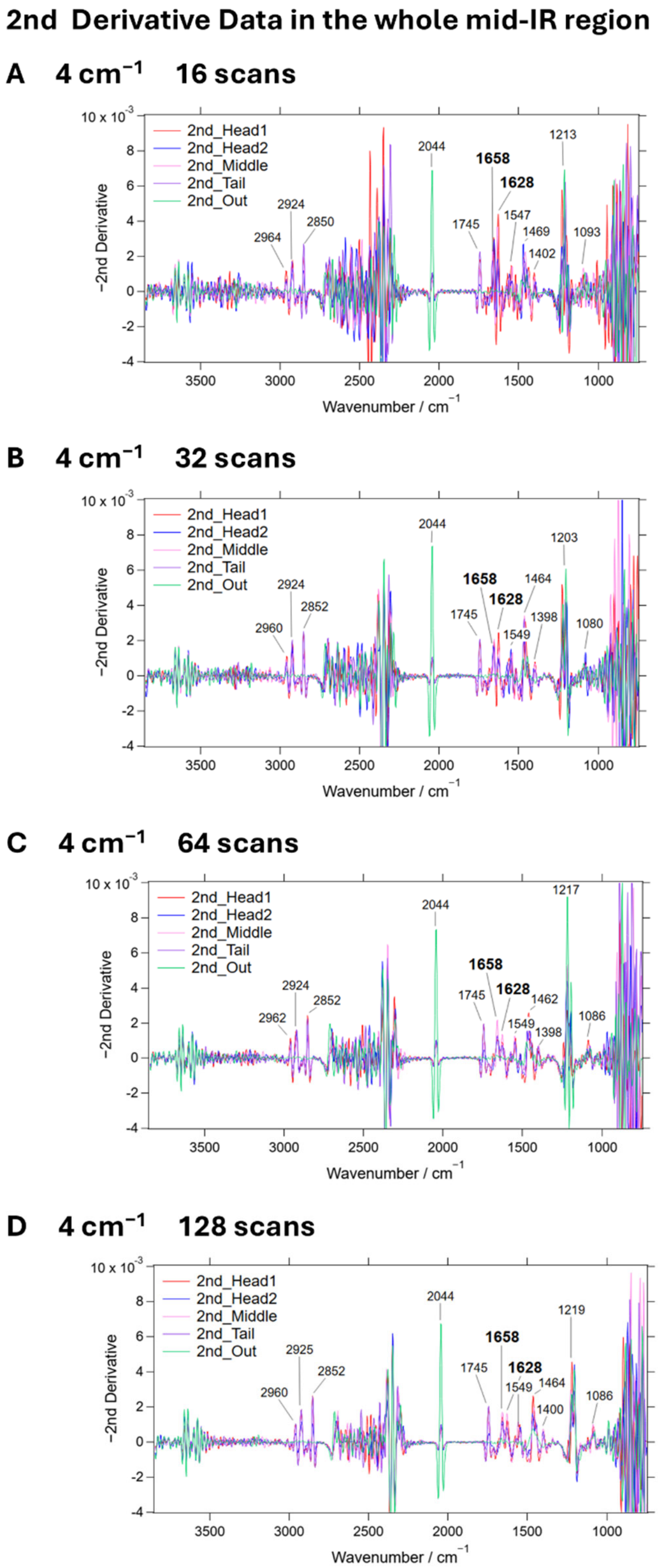

Figure S12. Comparison of 2nd derivative infrared absorption spectra from different positions of *C. elegans* in the whole mid-infrared region acquired at 4 cm^{-1} spectral resolution with 16, 32, 64, and 128 scans. Second-derivative infrared spectra were obtained from the following regions: red line (Head 1), blue line (Head 2), pink line (mid-body), purple line (tail), and green line (external solution). The exact sampling positions are shown as color-coded circles in Figure S10.

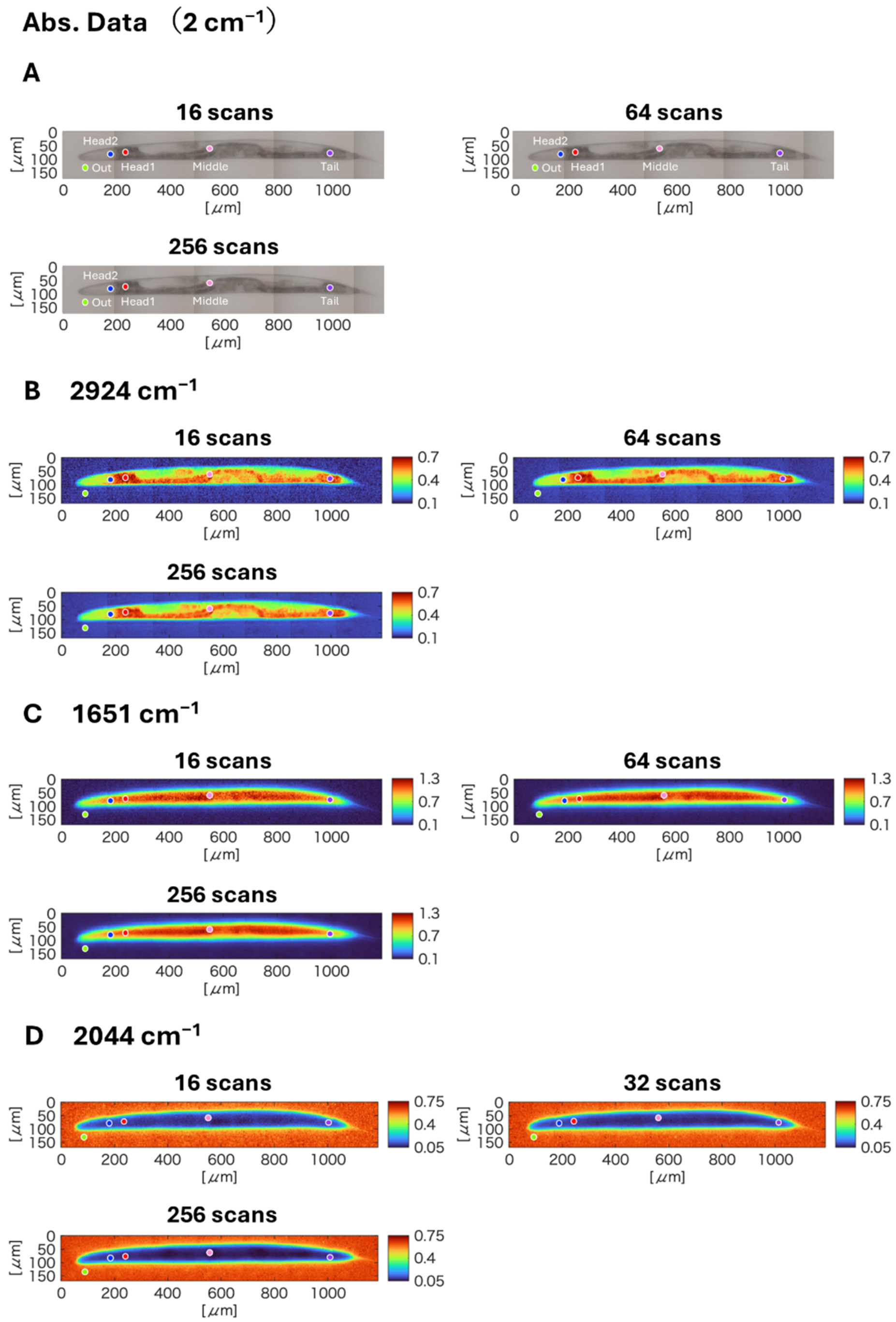

Figure S13. Comparison of infrared images acquired at 2 cm^{-1} spectral resolution with varying scan numbers (16, 64, and 256 scans) at 2924, 1651, and 2044 cm^{-1}. Infrared spectra obtained from the colored circles are shown in Figure S14 and S15 (Red circle: Head 1; blue circle: Head 2; pink circle: mid-body; purple circle: tail; green circle: external solution).

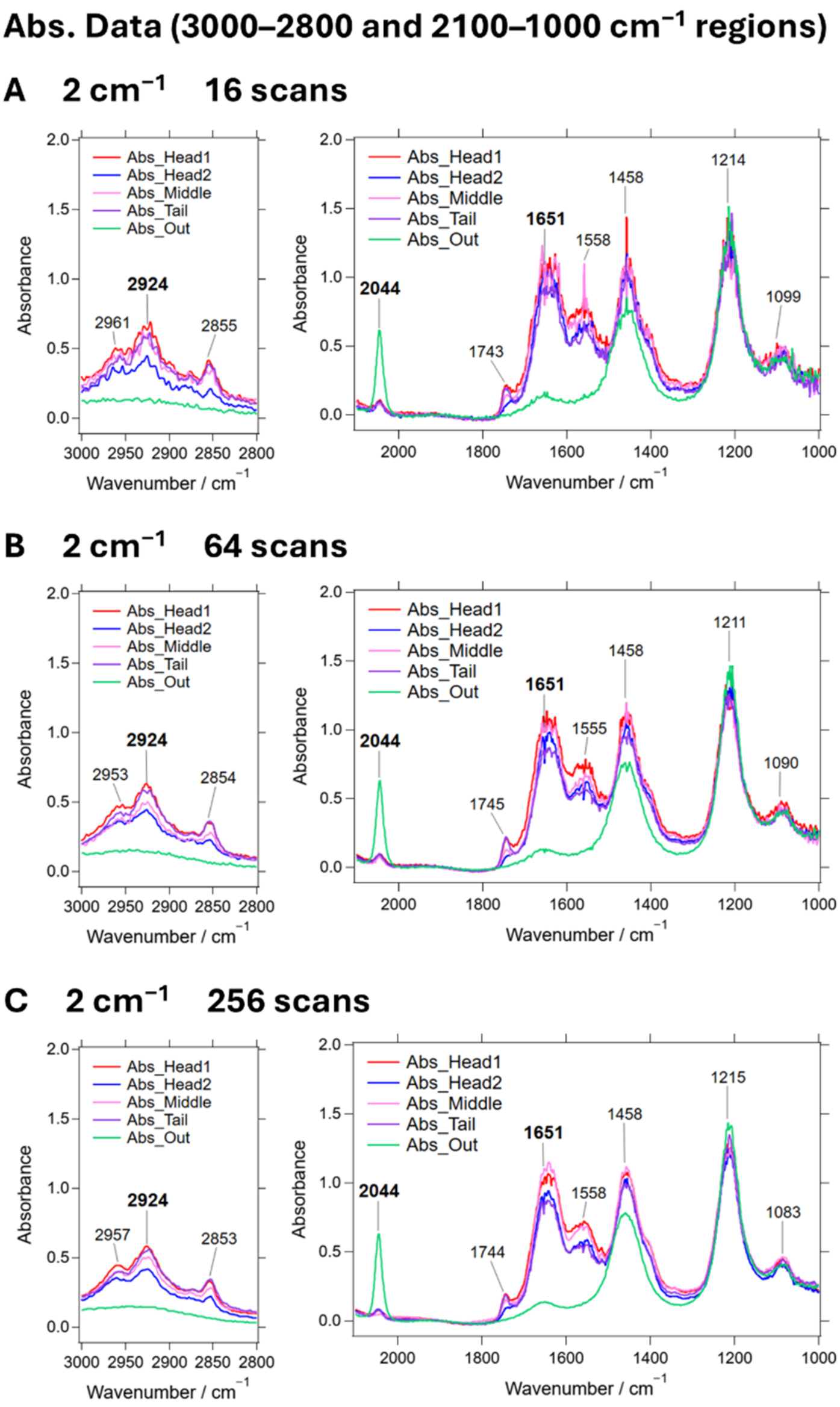

Figure S14. Comparison of infrared absorption spectra from different positions of *C. elegans* (3000–2800 cm^{-1} and 2100–1000 cm^{-1}) acquired at 2 cm^{-1} spectral resolution with 16, 64, and 256 scans. Infrared spectra were obtained from the following regions: red line (Head 1), blue line (Head 2), pink line (mid-body), purple line (tail), and green line (external solution). The exact sampling positions are shown as color-coded circles in Figure S13.

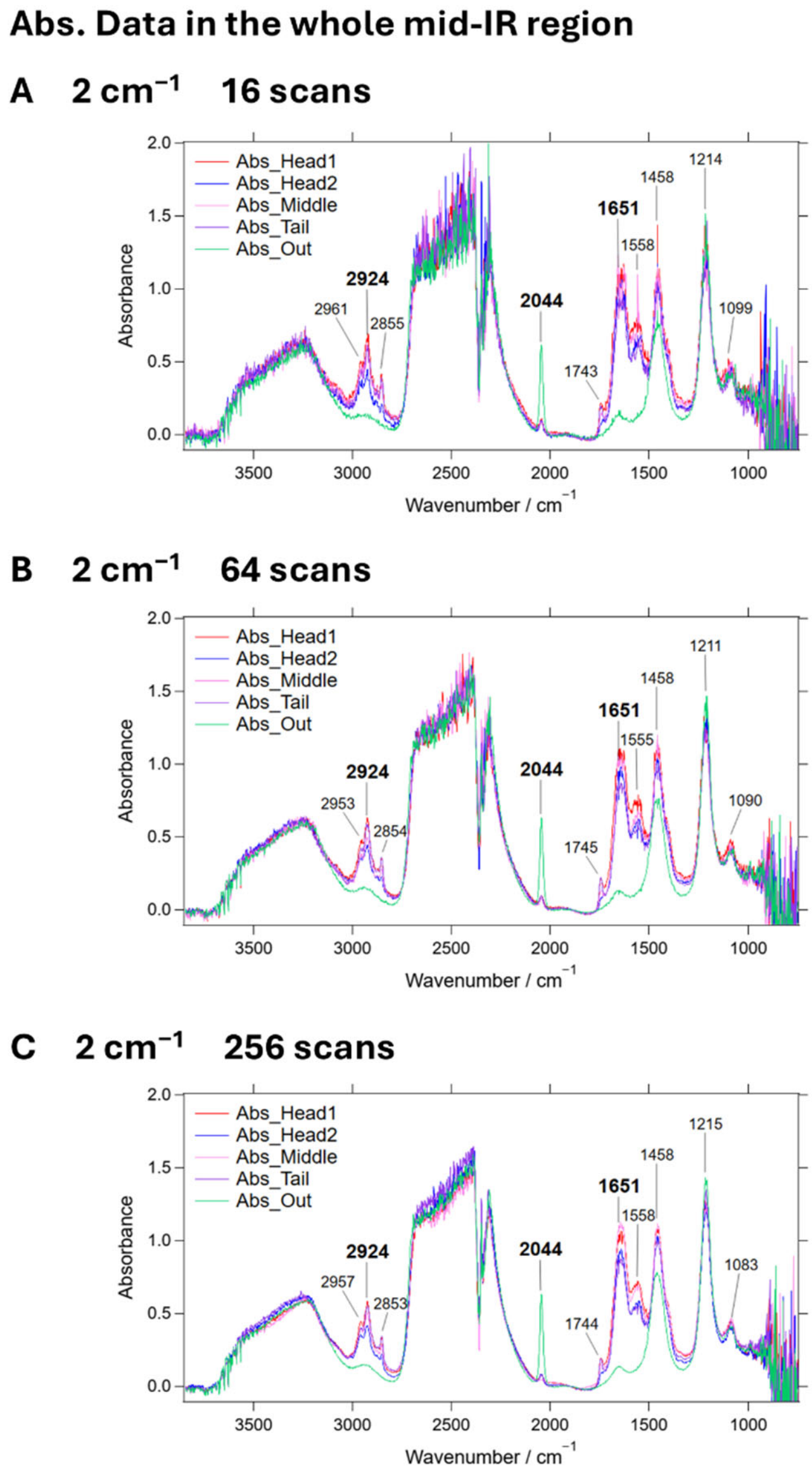

Figure S15. Comparison of infrared absorption spectra from different positions of *C. elegans* in the whole mid-infrared region acquired at 2 cm^{-1} spectral resolution with 16, 64, and 256 scans. Infrared spectra were obtained from the following regions: red line (Head 1), blue line (Head 2), pink line (mid-body), purple line (tail), and green line (external solution). The exact sampling positions are shown as color-coded circles in Figure S13.

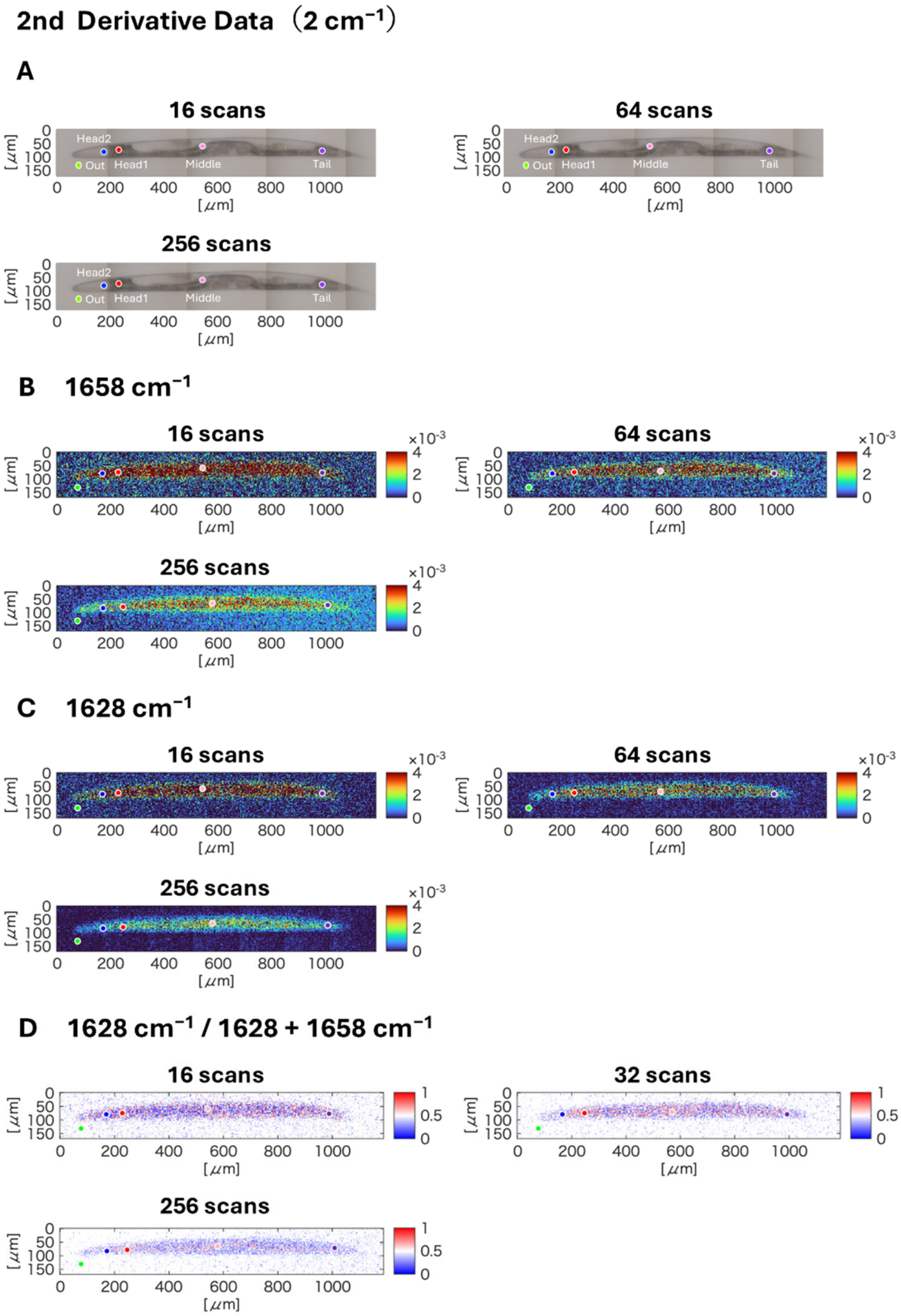

Figure S16. Comparison of 2nd derivative infrared images acquired at 2 cm^{-1} spectral resolution with varying scan numbers (16, 64, and 256 scans) at 1658, 1628 cm^{-1} and the ratio image at 1628 cm^{-1}/ 1628 + 1658 cm^{-1}. Second-derivative infrared spectra obtained from the colored circles are shown in Figure S17 and S18 (Red circle: Head 1; blue circle: Head 2; pink circle: mid-body; purple circle: tail; green circle: external solution).

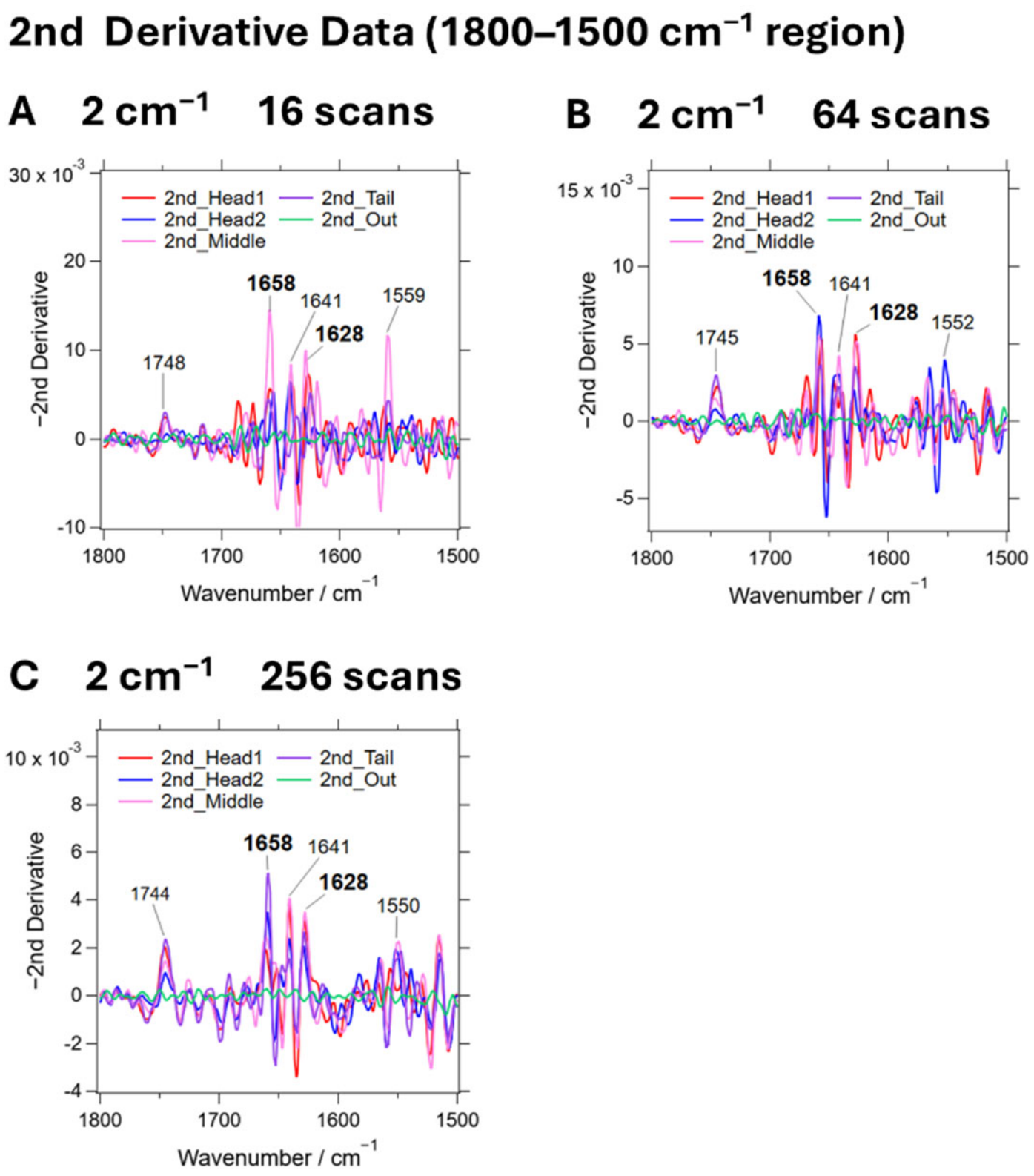

Figure S17. Comparison of 2nd derivative infrared absorption spectra from different positions of *C. elegans* (1800–1500 cm^{-1}) acquired at 2 cm^{-1} spectral resolution with 16, 64, and 256 scans. Second-derivative infrared spectra were obtained from the following regions: red line (Head 1), blue line (Head 2), pink line (mid-body), purple line (tail), and green line (external solution). The exact sampling positions are shown as color-coded circles in Figure S16.

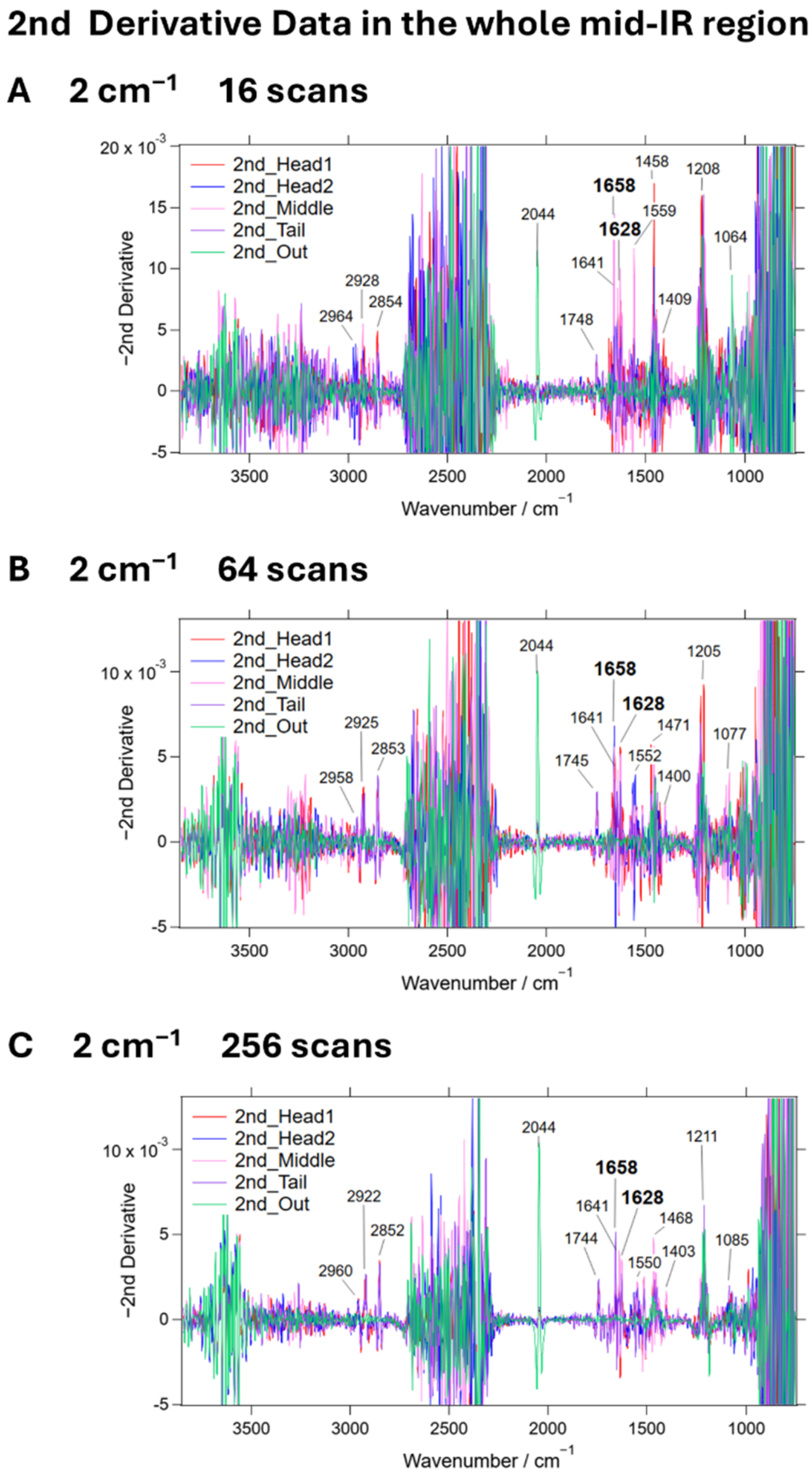

Figure S18. Comparison of 2nd derivative infrared absorption spectra from different positions of *C. elegans* in the whole mid-infrared region acquired at 2 cm^{-1} spectral resolution with 16, 64, and 256 scans. Second-derivative infrared spectra were obtained from the following regions: red line (Head 1), blue line (Head 2), pink line (mid-body), purple line (tail), and green line (external solution). The exact sampling positions are shown as color-coded circles in Figure S16.

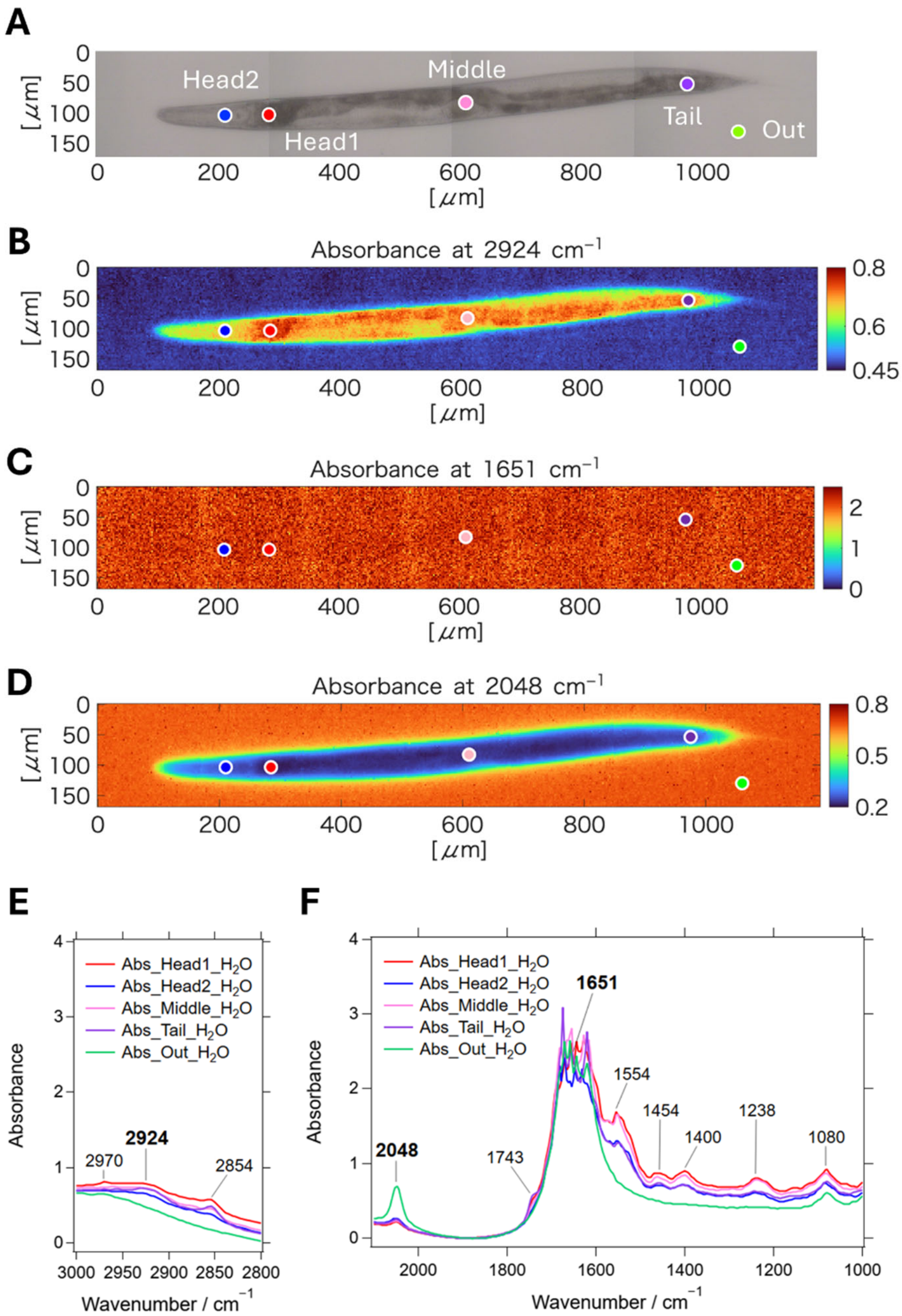

Figure S19. Infrared imaging of *C. elegans* performed in H_2O. (A) Optical micrograph of a living worm showing its morphology and measurement region. (B–D) Infrared absorbance images. (B) 2924 cm^{-1}: CH_2 stretching vibration derived from lipids. (C) 1651 cm^{-1}: Amide I vibration derived from proteins. (D) 2048 cm^{-1}: Absorption derived from the azide group. The X and Y axes indicate the worm scale (μm). The color bar on the right represents infrared absorbance intensity. (E, F) Representative infrared absorbance spectra obtained from different regions. (E) 3000–2800 cm^{-1} (CH stretching region of lipids). (F) 2100–1000 cm^{-1} (region containing azide and amide bands). Spectra were collected from five positions (Head1 = red, Head2 = blue, Middle = pink, Tail = purple, Out = green).

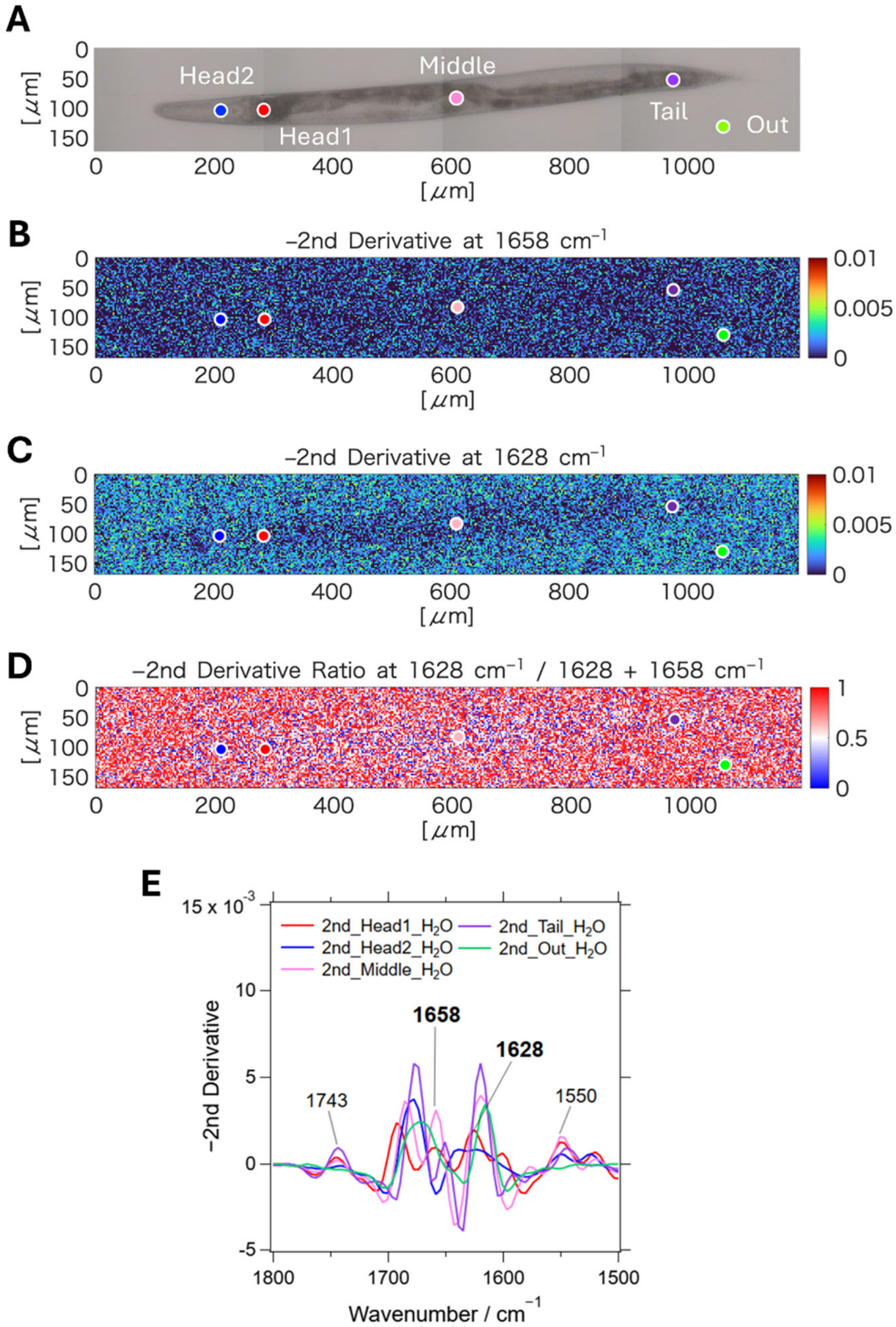

Figure S20. Infrared imaging with second derivative treatment of *C. elegans* performed in H_2O. (A) Optical micrograph of a living worm showing morphology and measurement region. (B, C) Second-derivative infrared absorbance images. (B) 1658 cm^{-1}: α-helix structure within the Amide I bands. (C) 1628 cm^{-1}: β-sheet structure within the Amide I bands. The right color bar indicates the second-derivative absorbance intensity. (D) Ratio image (1628 cm^{-1} / 1628 + 1658 cm^{-1}) showing relative distribution of α-helix and β-sheet components. The X and Y axes indicate the worm scale (μm). (E) Representative infrared absorbance spectra (1800–1500 cm^{-1}, including Amide bands) collected from five positions (Head1 = red, Head2 = blue, Middle = pink, Tail = purple, Out = green).

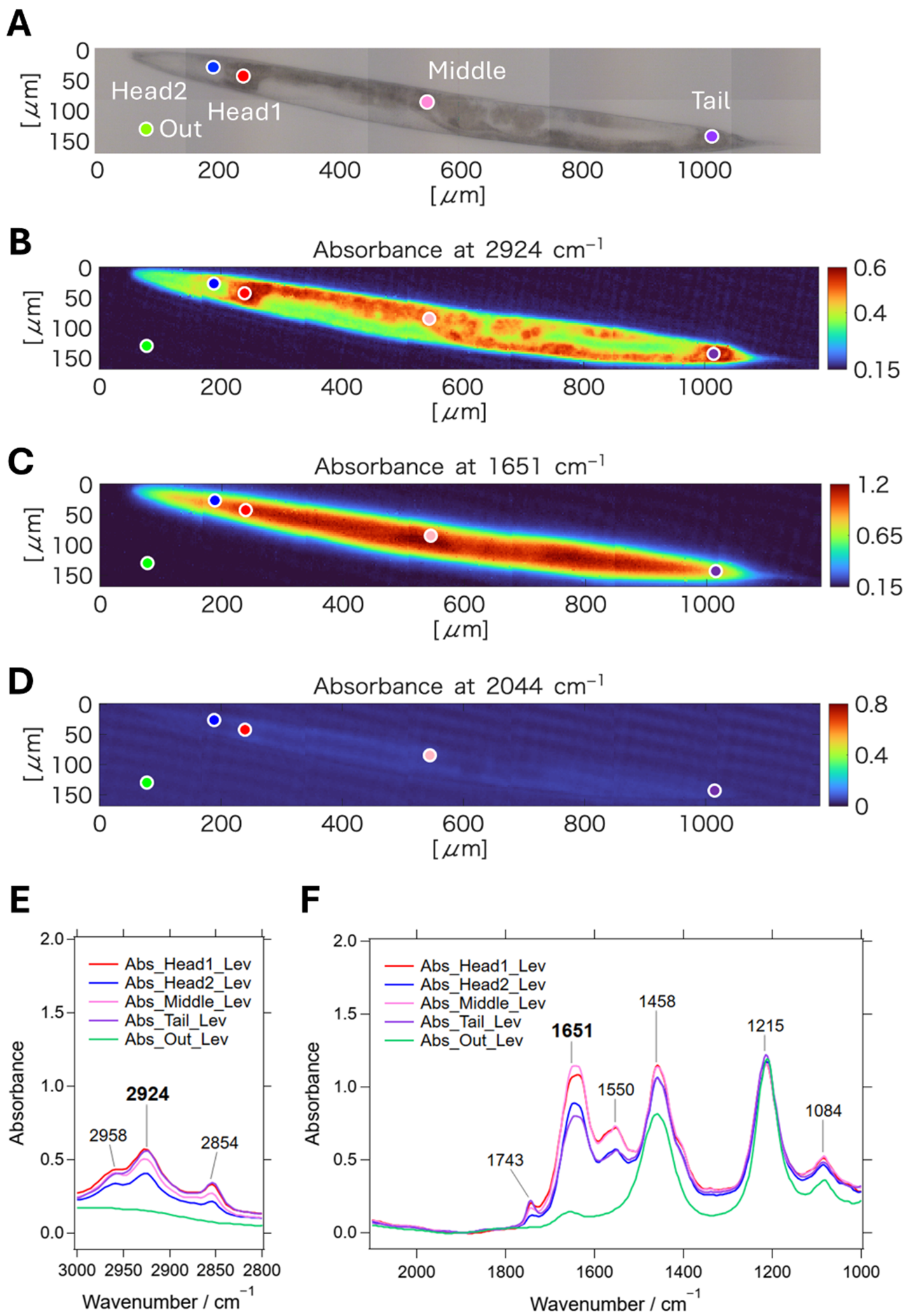

Figure S21. Infrared imaging of *C. elegans* immobilized by levamisole in D_2O. (A) Optical micrograph of a living worm showing its morphology and measurement region. (B–D) Infrared absorbance images. (B) 2924 cm^{-1}: CH_2 stretching vibration derived from lipids. (C) 1651 cm^{-1}: Amide I vibration derived from proteins. (D) 2044 cm^{-1}: Absorption derived from the azide group. The X and Y axes indicate the worm scale (μm). The color bar on the right represents infrared absorbance intensity. (E, F) Representative infrared absorbance spectra obtained from different regions. (E) 3000–2800 cm^{-1} (CH stretching region of lipids). (F) 2100–1000 cm^{-1} (region containing azide and amide bands). Spectra were collected from five positions (Head1 = red, Head2 = blue, Middle = pink, Tail = purple, Out = green).

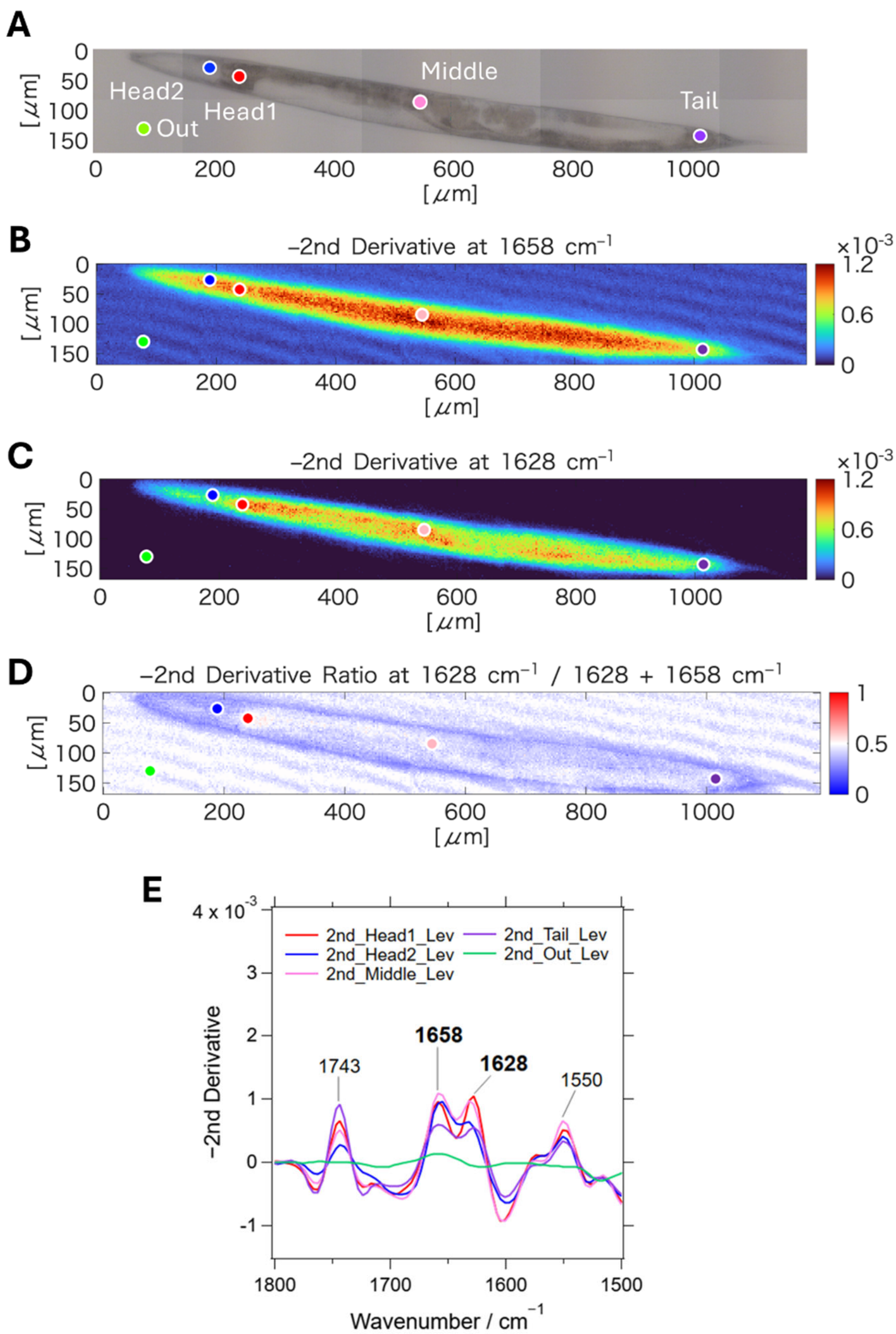

Figure S22. Infrared imaging with second derivative treatment of *C. elegans* immobilized by levamisole in D_2O. (A) Optical micrograph of a living worm showing morphology and measurement region. (B, C) Second-derivative infrared absorbance images. (B) 1658 cm^{-1}: α-helix structure within the Amide I bands. (C) 1628 cm^{-1}: β-sheet structure within the Amide I bands. The right color bar indicates the second-derivative absorbance intensity. (D) Ratio image (1628 cm^{-1} / 1628 + 1658 cm^{-1}) showing relative distribution of α-helix and β-sheet components. The X and Y axes indicate the worm scale (µm). (E) Representative infrared absorbance spectra (1800–1500 cm^{-1}, including Amide bands) collected from five positions (Head1 = red, Head2 = blue, Middle = pink, Tail = purple, Out = green).

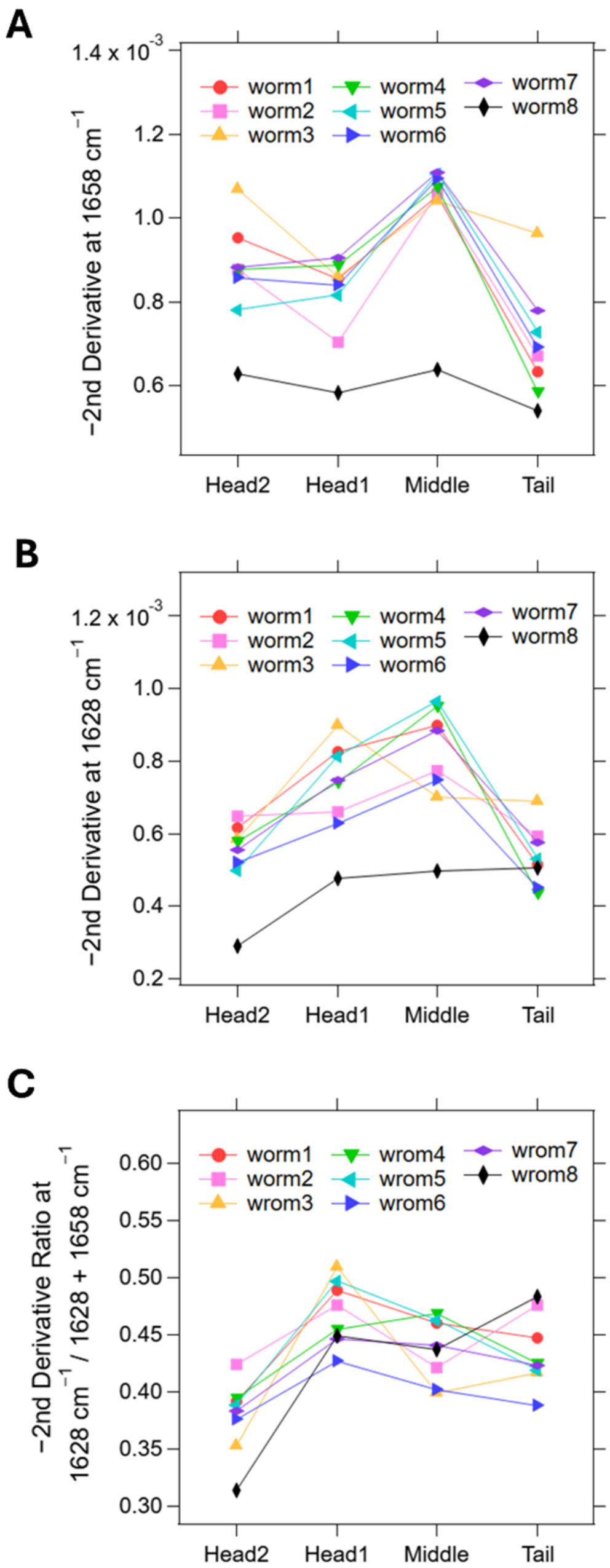

Figure S23. Line plots of second-derivative values at 1658 cm⁻¹ and 1628 cm⁻¹, and their ratio (1628 cm^{-1}/ 1628 + 1658 cm^{-1}) for eight individual worms. Data for each body region (Head2, Head1, Middle, and Tail) are shown using color-coded lines and symbols. The correspondence between colors and markers is indicated within the figure.

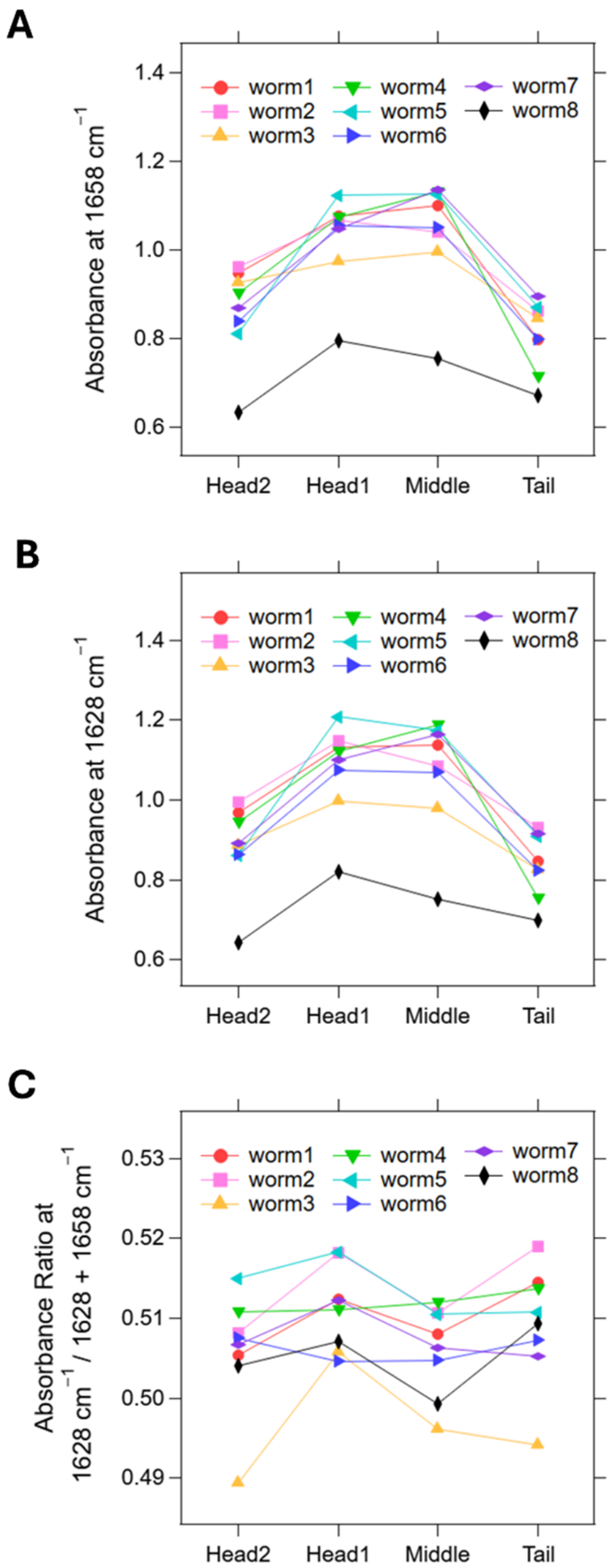

Figure S24. Line plots of infrared absorption values at 1658 cm^{-1} and 1628 cm^{-1}, and their ratio (1628 cm^{-1}/ 1628 + 1658 cm^{-1}) for eight individual worms. Data for each body region (Head2, Head1, Middle, and Tail) are shown using color-coded lines and symbols. The correspondence between colors and markers is indicated within the figure.

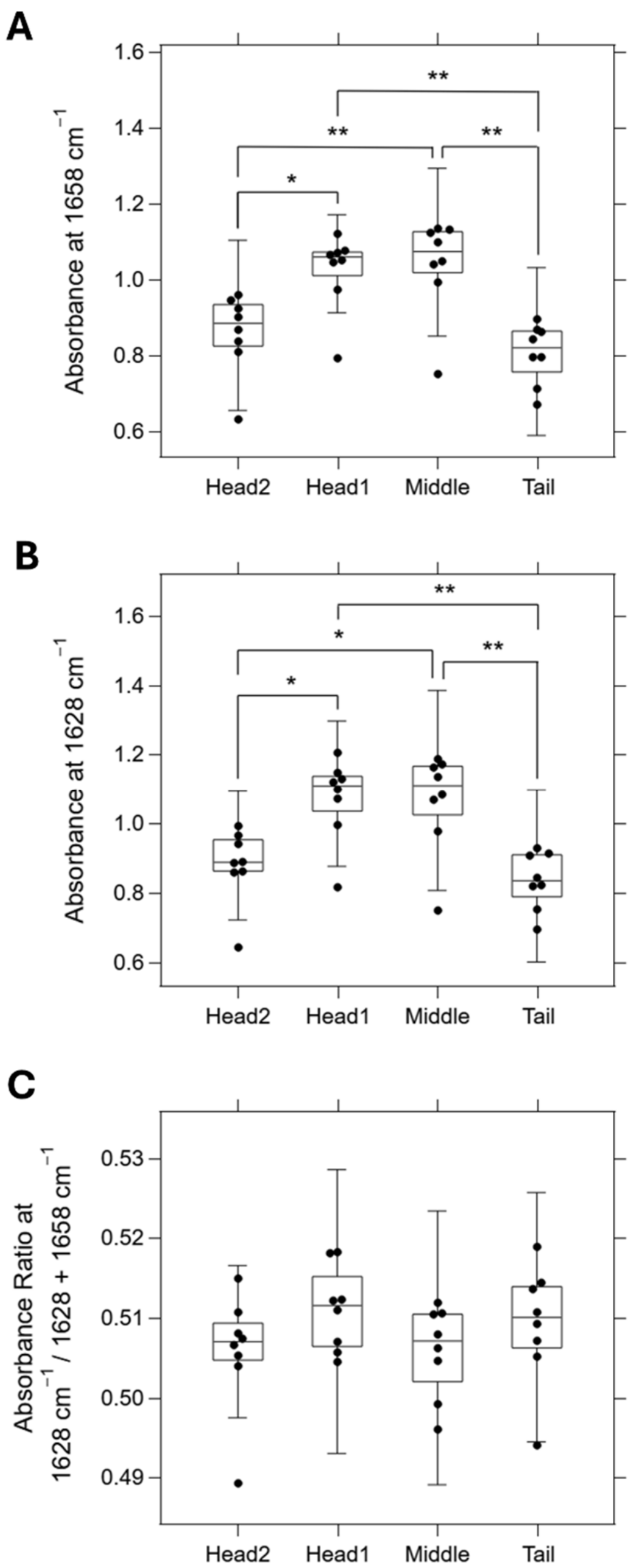

Figure S25. Box plots of the infrared absorption values for 1658 cm^{-1} (A) and 1628 cm^{-1} (B) and their ratio values (1628 cm^{-1}/ 1628 + 1658 cm^{-1}) (C) for eight worms. Each box represents the interquartile range (IQR), and whiskers extend to 1.5 × IQR. The median is indicated by a horizontal line within the box. Statistical significance determined by Tukey's test is indicated as * ($P < 0.05$) and ** ($P < 0.01$). Detailed results of Tukey's multiple comparison tests are provided in Tables S2–4.

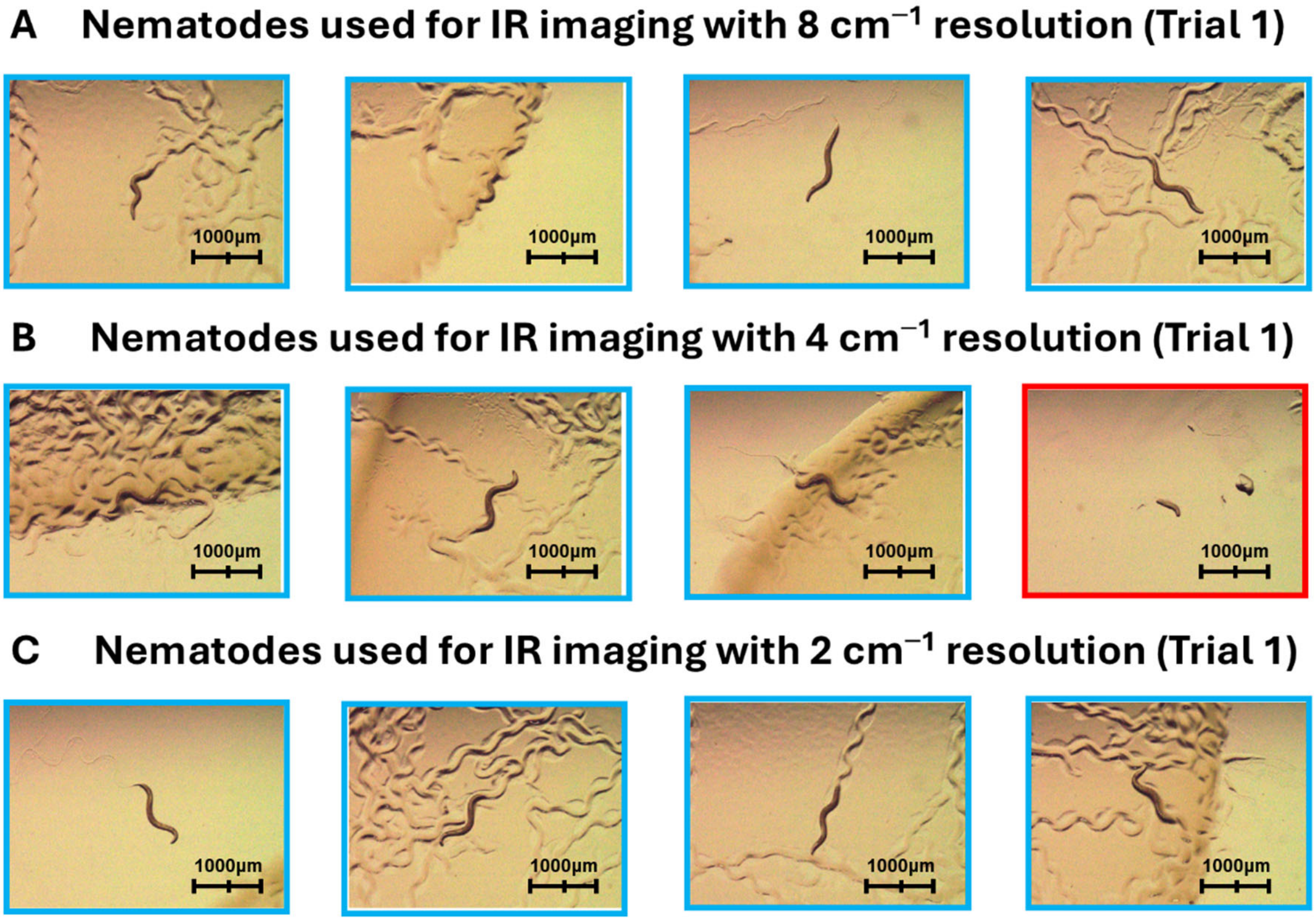

Figure S26. Representative images confirming survival of *C. elegans* after infrared imaging at 8, 4, and 2 cm^{-1} resolution. Worms surrounded by blue frames indicate successful recovery (resumption of movement), while those surrounded by red frames indicate failure to recover.

Table S1. Mean values of infrared absorbance with standard errors (SE) for the Head2, Head1, Middle, and Tail regions, obtained from eight worms.

Raw value	Head2		Head1		Middle		Tail	
	Mean	SE	Mean	SE	Mean	SE	Mean	SE
1658 cm^{-1}	0.861	0.038	1.026	0.036	1.042	0.045	0.807	0.028
1628 cm^{-1}	0.881	0.038	1.075	0.042	1.069	0.051	0.837	0.029
1628 cm^{-1} / 1628 + 1658 cm^{-1}	0.506	0.003	0.511	0.002	0.506	0.002	0.509	0.003

Table S2. Results of Tukey's multiple comparison test based on the absorbance of 1658 cm^{-1} shown in Table S1.

Pairwise comparisons among the regions are listed, along with the mean difference, q value, P value, and significance level. The q value represents the Studentized range statistic used in Tukey's multiple comparison test, and the P value indicates the probability associated with each pairwise comparison. Statistical significance is indicated as n.s. (not significant, $P > 0.05$), * ($P < 0.05$), and ** ($P < 0.01$).

Pairwise comparison	Difference	q value	P value	Significance
Head1 – Head2	0.165	4.46	0.0189	*
Middle − Head2	0.181	4.87	0.00924	**
Middle − Head1	0.015	0.414	0.991	n.s.
Head1 − Tail	0.219	5.92	0.00138	**
Head2 − Tail	0.054	1.46	0.733	n.s.
Middle − Tail	0.235	6.33	0.000632	**

Table S3. Results of Tukey's multiple comparison test based on the absorbance of 1628 cm^{-1} shown in Table S1.

Pairwise comparisons among the regions are listed, along with the mean difference, q value, P value, and significance level. The q value represents the Studentized range statistic used in Tukey's multiple comparison test, and the P value indicates the probability associated with each pairwise comparison. Statistical significance is indicated as n.s. (not significant, $P > 0.05$), * ($P < 0.05$), and ** ($P < 0.01$).

Pairwise comparison	Difference	q value	P value	Significance
Head1 − Head2	0.193	4.71	0.0122	*
Middle − Head2	0.187	4.56	0.0158	*
Head2 − Tail	0.044	1.07	0.872	n.s.
Head1 − Middle	0.006	0.15	1	n.s.
Head1 − Tail	0.237	5.79	0.00177	**
Middle − Tail	0.231	5.64	0.00233	**

Table S4. Results of Tukey's multiple comparison test based on the ratio of the absorbance of 1628 cm^{-1}/ 1628 + 1658 cm^{-1} shown in Table S1.

Pairwise comparisons among the regions are listed, along with the mean difference, q value, P value, and significance level. The q value represents the Studentized range statistic used in Tukey's multiple comparison test, and the P value indicates the probability associated with each pairwise comparison. Statistical significance is indicated as n.s. (not significant, $P > 0.05$).

Pairwise comparison	Difference	q value	P value	Significance
Head1 − Head2	0.00534	2.3	0.38	n.s.
Head2 − Middle	0.000091	0.04	1	n.s.
Tail − Head2	0.00339	1.46	0.731	n.s.
Head1 − Middle	0.00525	2.26	0.394	n.s.
Head1 − Tail	0.00195	0.84	0.933	n.s.
Tail − Middle	0.0033	1.42	0.747	n.s.

Table S5. Paired t-test results for the ratio (1628 cm^{-1}/ 1628 + 1658 cm^{-1}) between Head1 and Head2 without second-derivative processing.

Results of paired t-test comparing the ratio (1628 cm^{-1}/ 1628 + 1658 cm^{-1}) between Head1 and Head2 using raw absorbance values. The table includes the test type, number of samples (n), mean difference, t value, degrees of freedom (df), P value, and statistical significance. Statistical significance is indicated as * ($P < 0.05$).

Test	n	Mean difference	t	df	P value	Significance
Paired t-test	8	0.00534	2.53	7	0.0395	*

Table S6. Paired t-test results for the ratio (1628 cm^{-1}/ 1628 + 1658 cm^{-1}) between Head1 and Head2 after second-derivative processing.

Results of paired t-test comparing the ratio (1628 cm^{-1}/ 1628 + 1658 cm^{-1}) between Head1 and Head2 based on second-derivative values. The table includes the test type, number of samples (n), mean difference, t value, degrees of freedom (df), P value, and statistical significance. Statistical significance is indicated as *** ($P < 0.001$).

Test	n	Mean difference	t	df	P value	Significance
Paired t-test	8	0.09038	6.31	7	0.000399	***

Table S7. Experimental conditions for infrared imaging, including spectral resolution, number of scans, acquisition time, and data size.

Spectral resolution (cm^{-1})	Number of scans	Actual measurement time	Data size (MB)
8.0	16	3 min 30 s	92.2
8.0	32	4 min 10 s	92.2
8.0	64	6 min 47s	92.2
4.0	16	4 min 12 s	184.4
4.0	32	6 min 24 s	184.4
4.0	64	10 min 57 s	184.4
4.0	128	20 min 6 s	184.4
2.0	16	7 min 3 s	368.7
2.0	64	19 min 38 s	368.7
2.0	256	70 min 44 s	368.7

Table S8. Summary of post-imaging survival rates of *C. elegans* under different spectral resolution conditions.

Resolution of IR spectra	Number of worms used for IR imaging		Total number of worms	Number of surviving worms after IR imaging		Total number of surviving worms	Overall survival rate (%)
	(Trial 1)	(Trial 2)		(Trial 1)	(Trial 2)		
8 cm^{-1}	4	4	8	4	4	8	100
4 cm^{-1}	4	4	8	3	2	5	62.5
2 cm^{-1}	4	4	8	4	1	5	62.5

References

(1) Massie, M. R.; Lapoczka, E. M.; Boggs, K. D.; Stine, K. E.; White, G. E. Exposure to the metabolic inhibitor sodium azide induces stress protein expression and thermotolerance in the nematode *Caenorhabditis elegans*. *Cell Stress Chaperones* **2003**, *8* (1), 1-7.

(2) Manjarrez, J. R.; Mailler, R. Stress and timing associated with *Caenorhabditis elegans* immobilization methods. *Heliyon* **2020**, *6* (7), e04263.
